# Immune cell-resolved transcriptomics provides insights into the basis for variations of fish genetic resistance to viral disease

**DOI:** 10.1186/s12915-025-02452-z

**Published:** 2025-11-25

**Authors:** Thomas C. Clark, Valentin Thomas, Richard S. Taylor, Mathieu Charles, Audrey Laurent, Isabelle Schwartz-Cornil, Bertrand Collet, Delphine Lallias, Daniel J. Macqueen, Samuel A. M. Martin, Pierre Boudinot

**Affiliations:** 1 VIM, INRAE, UVSQ, Université Paris-Saclay, Jouy-en-Josas, 78350 France; 2 Scottish Fish Immunology Research Centre, School of Biological Sciences, University of Aberdeen, Aberdeen, AB24 2TZ UK; 3 GABI, INRAE, Université Paris-Saclay, Jouy-en-Josas, 78350 France; 4 The Roslin Institute and Royal (Dick) School of Veterinary Studies, University of Edinburgh, Edinburgh, UK; 5 LPGP, INRAE, Rennes, 35042 France

**Keywords:** VHSV, Genetic resistance to viral infections, Rainbow trout, Interferon, Inflammation

## Abstract

**Background:**

The genetic basis of host resistance to viral infections is generally shaped by complex interactions between host genetic variations affecting antiviral immunity and the rapid evolutionary adaptability of viruses. In this study, we investigated two isogenic rainbow trout lines exhibiting extreme resistance or susceptibility to the rhabdovirus VHSV. We compared transcriptomes of the pronephros — a major lymphoid organ in fish — at steady state and following VHSV infection. By integrating bulk tissue RNA-seq with single-cell RNA-seq, we mapped the divergent transcriptomic responses of resistant and susceptible fish to specific immune cell types.

**Results:**

At steady state, differences in antiviral pathways were minimal. However, VHSV triggered markedly distinct transcriptomic shifts between the lines. Both resistant and susceptible fish exhibited a broad transcriptional response enriched in core type I interferon (IFN) pathway genes. However, line-specific responses were enriched in genes induced by infection independently of type I IFN. In resistant fish, lymphocyte responses included type I IFN pathway, numerous transcription factors, and various cytokine receptors. In contrast, lymphocyte responses in susceptible fish involved only a limited set of type I IFN-induced genes. Monocytic cell responses also diverged: susceptible fish upregulated IFN-induced genes, while resistant fish showed increased expression of proinflammatory genes.

**Conclusions:**

This study reveals the contribution of the core set of interferon-stimulated genes conserved across vertebrates to the response of different immune cells and the response of other genes in resistant and susceptible fish. It provides a comprehensive basis for evolutionary studies of resistance to viruses in vertebrates.

**Supplementary Information:**

The online version contains supplementary material available at 10.1186/s12915-025-02452-z.

## Background

The outcome of any infection is determined by the susceptibility of the host and the virulence of the pathogen. A number of host defence mechanisms limit pathogen multiplication and spread, including inflammation, which has side effects that are often deleterious to the host. The virulence of pathogens determines their capacity for replication and host invasion, leading to lesions and tissue damage. Thus, host–pathogen interactions are shaped by the coevolution of microbe virulence factors and host defence mechanisms. The heterogeneity of pathobiomes and the complexity of immune responses therefore lead to significant variation in resistance/susceptibility to pathogens within and between host populations [1].

The viral hemorrhagic septicemia virus (VHSV), a *Novirhabdovirus*, causes a severe haemorrhagic disease in farmed rainbow trout (*Oncorhynchus mykiss*) and in a number of wild fish species [2]. VHS is listed as a notifiable disease by the World Organization for Animal Health (WOAH) and can lead to high mortality rate, especially in sexually immature fish. VHSV is a single-stranded RNA virus with a non-segmented genome encoding five structural proteins and a nonstructural protein (NV) specific to genus *Novirhabdovirus* [3]. The base of the fins is the major portal of entry for fish [4], and fibronectin has been identified as the receptor of VHSV [5]. The virus replicates mainly in epithelial and fibroblastic cells, causing a systemic infection. Genetic resistance in rainbow trout to VHSV has been reported with high heritability in independent studies: 0.57 in [6] and 0.63 in [7]. Indeed, a wide range of susceptibility to VHSV infection has been observed across a collection of rainbow trout isogenic lines, underlining the key role of genetics [8]. Studies of rainbow trout families identified several survival-associated quantitative trait loci (QTL) following VHSV infection, including a major-effect QTL on chromosome 3 and smaller-effect QTLs on several others [9, 10].

The correlation between VHSV growth on fin explants and host viral susceptibility previously supported a hypothesis that resistance mechanisms are intrinsic/innate, rather than linked to a systemic adaptive response [11, 12]. Consistent with this, all known resistance QTL are not located in genomic regions containing major histocompatibility complex (MHC) class I or class II genes. This hypothesis was further supported by the perfect correlation between the susceptibility of isogenic doubled haploid rainbow trout lines to infection and the susceptibility of fibroblast-like cells derived from these fish [13]. Furthermore, induction of an early type I interferon (IFN) response in this resistant cell line, compared to a highly susceptible cell line, suggested that factors in the IFN pathway may underpin susceptibility to VHSV. The transcriptomic response induced by VHSV in these two cell lines was previously compared following standardised stimulation using inactivated VHSV. A stronger type I IFN response was observed in cells derived from the resistant fish compared to the susceptible line [10]. These data likewise point to the importance of the type I IFN response for VHSV resistance. However, different mechanisms of resistance — defined as a survival rate to waterborne infection — appeared to be present: while one of the rainbow trout isogenic lines was fully resistant to viral infection, other lines allowed virus replication but showed no mortality [8, 13]. Among susceptible lines showing high mortality upon bath infection, multiple kinetics of mortality also suggested differences in mechanisms of viral spreading and disease progression [8].

In this study, we compared the transcriptomic response to VHSV infection in a lymphoid organ of resistant (B57) and susceptible (AP2) isogenic rainbow trout lines, both at the whole tissue and single-cell level. These fish lines were produced using a gynogenesis-based strategy [8]. All individuals of a line are homozygous at all loci and therefore share identical MHC class I and class II haplotypes, immunoglobulin, and T-cell receptors loci, as well as the same repertoire of genes involved in innate immunity. While juvenile B57 fish are fully resistant to VHSV infection by immersion, AP2 fish of the same age showed a high degree of susceptibility [8, 14]. Both AP2 and B57 were susceptible to VHSV infection by injection, with similar clinical signs, indicating that the virus can replicate in both albeit inducing different transcriptome modifications and immune responses. The head kidney was selected among lymphoid organs for several reasons: (1) It is both a haematopoietic and lymphopoietic tissue in fish, in which B-cell differentiation takes place; (2) it contains diverse immune cell types from myeloid, lymphoid, and erythroid lineages; and (3) we have previously characterised the transcriptome response of this tissue to the viral RNA mimic poly(I:C) in vivo and ex vivo in non-clonal rainbow trout [15], which provides a reference for analysis. We monitored responses of head kidney in fish from susceptible (AP2) and resistant (B57) lines after VHSV injection and also compared head kidney transcriptomes at steady state prior to infection. To obtain comprehensive insights into the differences between head kidney transcriptomes of susceptible and resistance genotypes, we combined bulk RNA-seq of head kidney tissue and single-cell RNA-seq (scRNA-seq) of purified head kidney leukocytes. Several recent reports of scRNA-seq of spleen and head kidney-derived leukocytes from salmonids have revealed marked diversity of subsets in the immune cellular repertoire and their distinctive specific markers [16, 17]. Analysis of rainbow trout B-cell subsets from peripheral blood leucocytes (PBLs) further illustrated the challenge of annotating cell types defined by scRNA-seq in salmonids, where cell subpopulations are poorly characterised compared to human or mice [18]. After infection with infectious pancreatic necrosis virus (IPNV), head kidney scRNA-seq revealed extensive modifications in myeloid cell gene expression, including monocytes/macrophages and neutrophils, compared to lymphoid subsets of PBLs [19].

The current study of rainbow trout lines susceptible and resistant to VHSV bridges the gap between genomic data obtained from QTL or genome-wide association studies and the biological interpretation of genetic resistance to viral infection. Here, we reveal a striking contrast of head kidney lymphoid and myeloid cell responses between resistant and susceptible isogenic rainbow trout, identifying potential cellular mechanisms involved in both innate and adaptive immunity offering resistance to a virus.

## Results

We compared the transcriptome response of head kidney from susceptible AP2 and resistant B57 rainbow trout lines to VHSV infection. We used fully developed sexually immature fish, in which the adaptive immune system and all immune cells types are functionally mature. Transcriptome analyses were performed 72 h after infection, when the type I IFN response is fully active. We combined bulk whole head kidney tissue RNA-seq and scRNA-seq analysis of purified leukocytes to enable a higher depth of sequencing and resolve responses of immune cell types present in the head kidney. The rationale of this approach was to investigate the differences in cell composition and transcriptome expression between resistant and susceptible fish before and after viral infection.

### B57 and AP2 head kidney transcriptomes at steady state disclose subtle differences

**Fig. 1:**
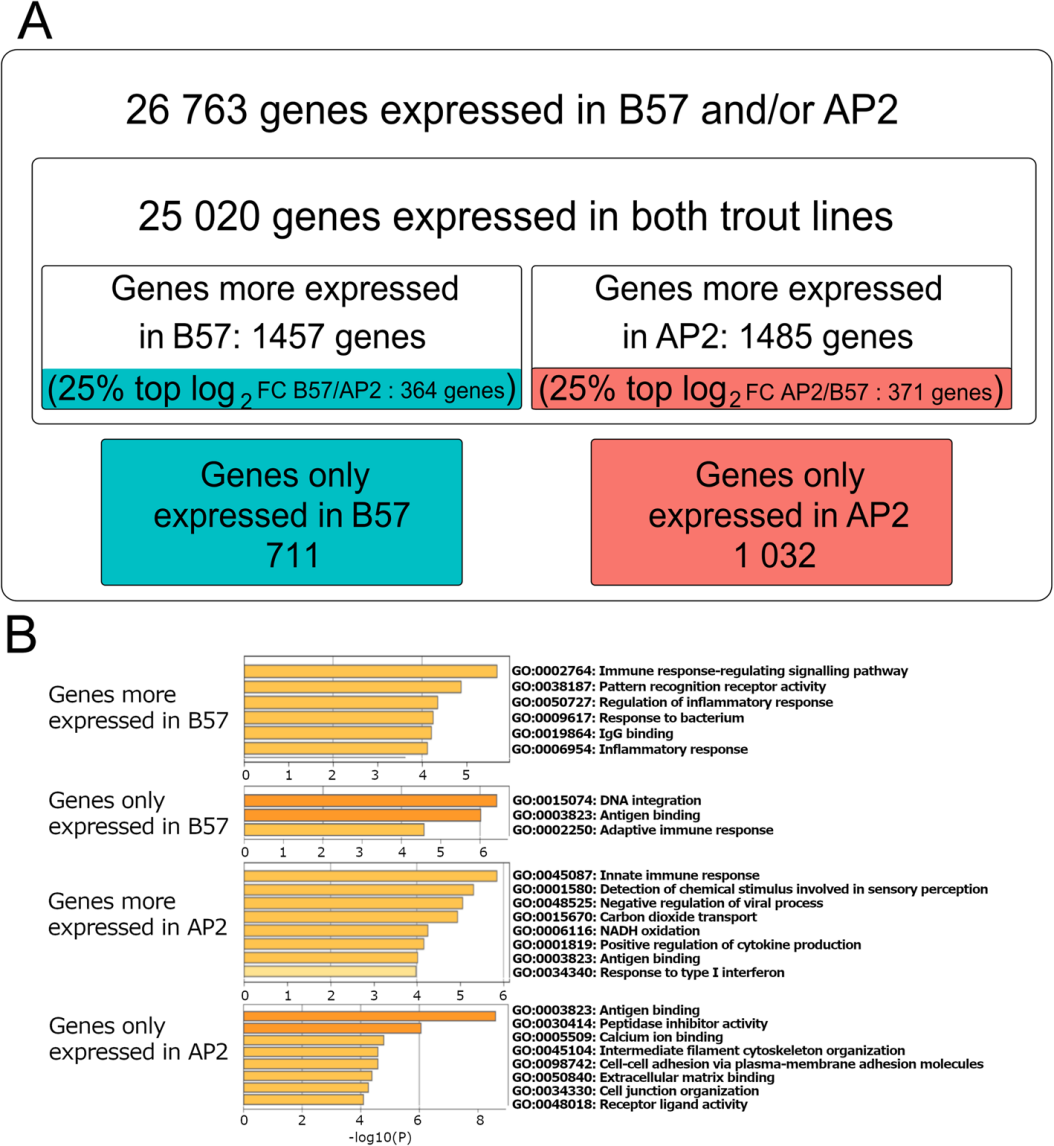
Transcriptome analysis of B57 and AP2 head kidney tissue at steady state. **A** Number of genes expressed in B57 and/or AP2 head kidney tissue (raw counts > 10) is shown. Differential analysis with deseq2 provided the numbers of genes expressed in both rainbow trout lines, of genes more expressed in one line (adjp < 0.01), and of genes with the most contrasted levels (25% top expression ratio between AP2 (in red) and B57(in blue), adjp < 0.01). The numbers of genes expressed only in AP2 (in red) or B57 (in blue) (raw counts > 10) are also indicated. **B** Gene Ontology enrichment analysis with Metascape for lists of genes expressed only or mostly (top 25% |Log2FC(AP2/B57)|) in either B57 or AP2. Full annotated lists of differentially expressed genes between AP2 and B57 head kidney are available in Additional File 1. Full Metascape results are available in Additional File 2

We started by comparing the global transcriptome of head kidney from control AP2 and B57 samples. About half of the repertoire of rainbow trout genes were expressed in this tissue: 25,731 and 26,052 respective genes in B57 and AP2, with 25,020 common to both (Fig. 1A; Additional File 1). Differential analysis revealed that 1457 genes were more highly expressed in B57 than AP2, while 1485 were more highly expressed in AP2 (Fig. 1A). The top 25% of genes with the highest fold change values in each line (i.e. 364 genes (FC B57/AP2 > = 3.17) for B57 and 371 (FC AP2/B57 > = 3.41) for AP2) were selected for Gene Ontology enrichment analysis in addition to genes expressed in only 1 rainbow trout line (Fig. 1B, Additional File 2). Genes with contrasted expression in AP2 and B57 were primarily associated with functions related to immune and inflammatory responses (Fig. 1B). Both lists were enriched in genes involved in “antigen binding”, including several genes encoding Ig (immunoglobulin) light chains, T-cell receptor, and MHC proteins (class 1, class 2, and B2M). This GO term reflected differences in Ig/TCR expressed genes and MHC haplotypes. In contrast, genes more expressed in B57 were linked to the term “immune response-regulating signalling pathway” (GO:0002764), based on the presence of caspase 1, several NOD-like receptor genes (*nod2*, *nlrps*, *nlrcs*), and toll-like receptors (*tlr2* and *3*). Genes more expressed in AP2 were enriched in genes involved in “negative regulation of viral processes” (GO:0048525), including a number of key interferon-stimulated genes (ISGs) such as *mx1*, *ifitm3*, *ifit5*, *rsad2*, and *trim25* [15]. In addition to core-conserved ISGs, B57- and AP2-specific gene lists also included genes associated with the “antigen binding” (GO:0002250), such as MHC (class 1, class 2, and B2M). All four lists contained genes involved in the “innate immune response” (GO:0045087). Genes expressed at similar levels in both fish lines were mostly associated with GO terms related to metabolic processes (GO:0008152). Overall, the GO analysis indicated that genes specific to, or more highly expressed in, a given genetic background may be enriched in immune-related genes with higher constitutive expression.

To build on this finding, we compared the expression level of all rainbow trout ISGs of the evolutionary conserved “core” repertoire defined previously [15], between AP2 and B57 head kidney at steady state (Additional File 3). Thirty percent of these ISGs were differentially expressed between AP2 and B57 (58 expressed more or only in AP2, 9 expressed more or only in B57, with *FC* > 2 and *adjp* < 0.01%), while the remaining 70% did not show differences in expression between the resistant and susceptible genetic backgrounds.

### scRNA-seq reveals similar repertoire of immune cells in AP2 and B57 head kidney at steady state

**Fig. 2:**
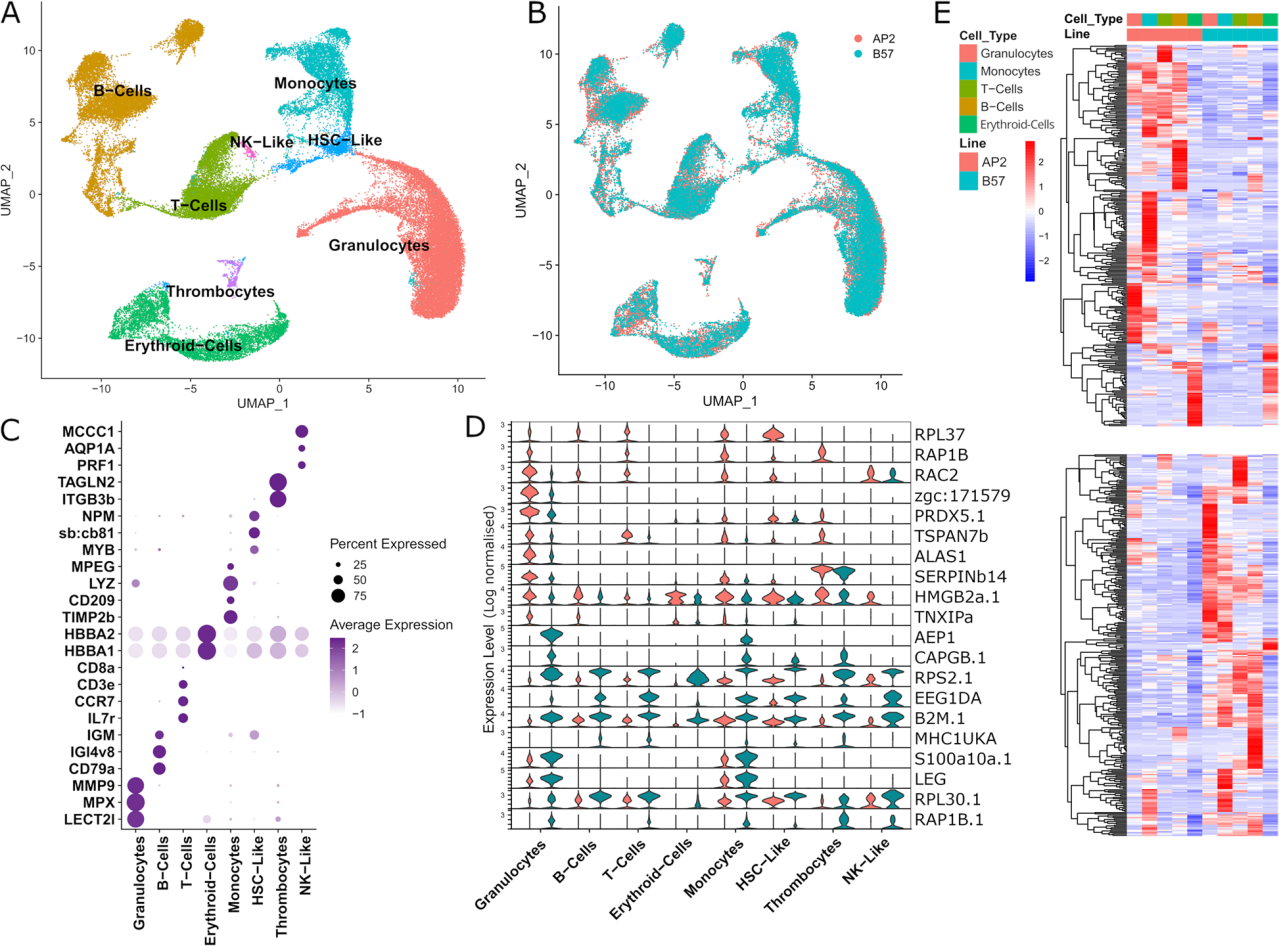
scRNA-seq analysis of AP2 and B57 head kidney immune cells at steady state. **A** UMAP (Uniform Manifold Approximation and Projection) of major cell types and lineages represented by different colours identified from joint Seurat analysis of all single-cell transcriptomes from AP2 and B57. **B** UMAP coloured by isogenic line, AP2 in red and B57 in blue. **C** Dot plot of marker genes identifying each major cell type/lineage, and dot size corresponds to the percentage of cells in a cluster expressing each marker and colour intensity representing level of expression. **D** Violin plots of genes with the top 10 highest fold change between AP2 and B57. **E** Heatmaps of differentially expressed genes, with top 25% logFC identified from RNA-seq deseq2 bulk analysis of AP2 and B57 head kidney at steady state (top heatmap, genes more expressed in AP2; bottom heatmap, genes more expressed in B57) projected onto the scRNA-seq dataset. Genes were filtered to only show those above 10 counts in the scRNA-seq dataset

We next compared the repertoire of immune cells purified from head kidney in AP2 and B57 using scRNA-seq. Transcriptomes of 52,829 head kidney cells were captured, with clustering identifying 8 main cell populations (Fig. 2A) defined by combinations of canonical markers [16, 17, 19] (Fig. 2B; Additional File 4a). The proportions of cells from each major lineage were very similar for AP2 and B57, barring monocytes (~ 16% and 8% of all immune cells in AP2 and B57, respectively) (Table 1; Additional File 4b). We then performed pseudobulk analysis, comparing transcriptomes of each major cell population grouped together between AP2 vs B57 (Additional File 4c for full differential gene lists). Top differentially expressed genes (i.e. genes with highest fold changes and adj *p*val < 5%) are shown by violin plots in Fig. 2D. These top differential genes between AP2 and B57 either showed contrasted expression across all cell types or highly different levels specifically in one or a few cell lineages. For example, aerolysin-like *aep1*, encoding a pore-forming protein, the most differentially expressed gene between the two lines [log2FC 3.6, gene expressed in 46% of B57 cells and 0.5% of AP2 cells], showed a highly specific pattern restricted to granulocytes and monocytes (Fig. 2D). *aep1* encodes a proinflammatory factor inducing *IL-1b* and *TNF-α* promoting antibacterial responses and pathogen clearance [20]. *s100a10*, which controls responsiveness to TLR stimulation by interfering with the recruitment of receptor proximal adaptors and determines the susceptibility to endotoxins [21], had a similar profile to *aep1*, with a strong enrichment in B57 cells. In contrast, *b2m.1* and the nonclassical MHC gene *mhc1uka* had highly contrasted expression between the two lines in all cell types (Fig. 2D), suggesting a general impact on peptide presentation mechanisms. Other highly contrasted genes included Rho GTPase encoding *rac2* and gelsolin encoding *capgb.1* (involved in actin capping), both playing a putative role in phagocytosis and macrophage biology [22, 23]; other genes were involved in translation (*rps2.1*, *rpl37*, *rpl30.1*, *rps28*, and *eef1da*) or were without a direct connection to defence mechanisms.

**Table 1:** Cell lineage proportions of AP2 and B57 fish at steady state and following VHSV infection (numbers of cells and percentage ± SEM)

	AP2	B57	VHSV AP2	VHSV B57
Granulocytes	9307 (33.2% ± 2.2%)	8826 (35.6% ± 2.4%)	1091 (35.8% ± 1.8%)	2634 (24.2% ± 7.3%)
B cells	6359 (22.7% ± 1.3%)	4845 (19.5% ± 2.2%)	896 (29.4% ± 1.2%)	3352 (30.8% ± 6.8%)
T cells	5103 (18.2% ± 2.2%)	3221 (13% ± 1.4%)	493 (16.2% ± 1.6%)	1518 (13.9% ± 2.8%)
Erythroid cells	3754 (13.4% ± 3.6%)	2860 (11.5% ± 1.8%)	194 (6.4% ± 0.8%)	1628 (15% ± 5.2%)
Monocytes	2267 (8.1% ± 0.7%)	3965 (16% ± 4.0%)	283 (9.3% ± 1.5%)	999 (9.2% ± 6.7%)
Other cell types	717 (2.6% ± 0.8%)	797 (3.2% ± 0.4%)	92 (3.0% ± 0.3%)	169 (1.6% ± 0.4%)

### scRNA-seq and RNA-seq datasets share similar expression profiles

Figure 2E shows that most genes with contrasted expression between AP2 and B57 RNA-seq derived from head kidney tissue have similar profiles to those from purified leukocytes scRNA-seq. From the genes highlighted in Fig. 1A, i.e. top 25% by fold change, we identified 323/371 and 343/364 genes in the scRNA-seq data for AP2 and B57 respectively with gene expression counts > 10 (Additional File 4d). Only 38 AP2 and 42 B57 genes showed an expression profile inconsistent with that observed in RNA-seq. Interestingly, many of the genes upregulated in one fish line were specific to one cell type in the case of AP2 or shared between either lymphocytes (T and B cells) or myeloid cells (granulocytes and monocytes) lineages in the case of B57. This result highlights the power of resolution offered by scRNA-seq, identifying the specific cell types responsible for differences observed in bulk RNA-seq.

Importantly, the concordance between gene sets with contrasted expression between AP2 and B57 in RNA-seq and scRNA-seq data indicated that they were mainly expressed in purified leukocytes.

### Comparison of AP2 and B57 immune cell subset transcriptomes identified no major functional shift between AP2 and B57

To fully harness the power offered from our scRNA-seq dataset, we next performed detailed comparisons of AP2 and B57 after additional clustering to reveal candidate subsets within each major immune population. The main findings for each immune cell lineage are summarised below, while the detailed analysis for all populations can be found in Additional File 5 [16, 24–52].

### T cells

**Fig. 3:**
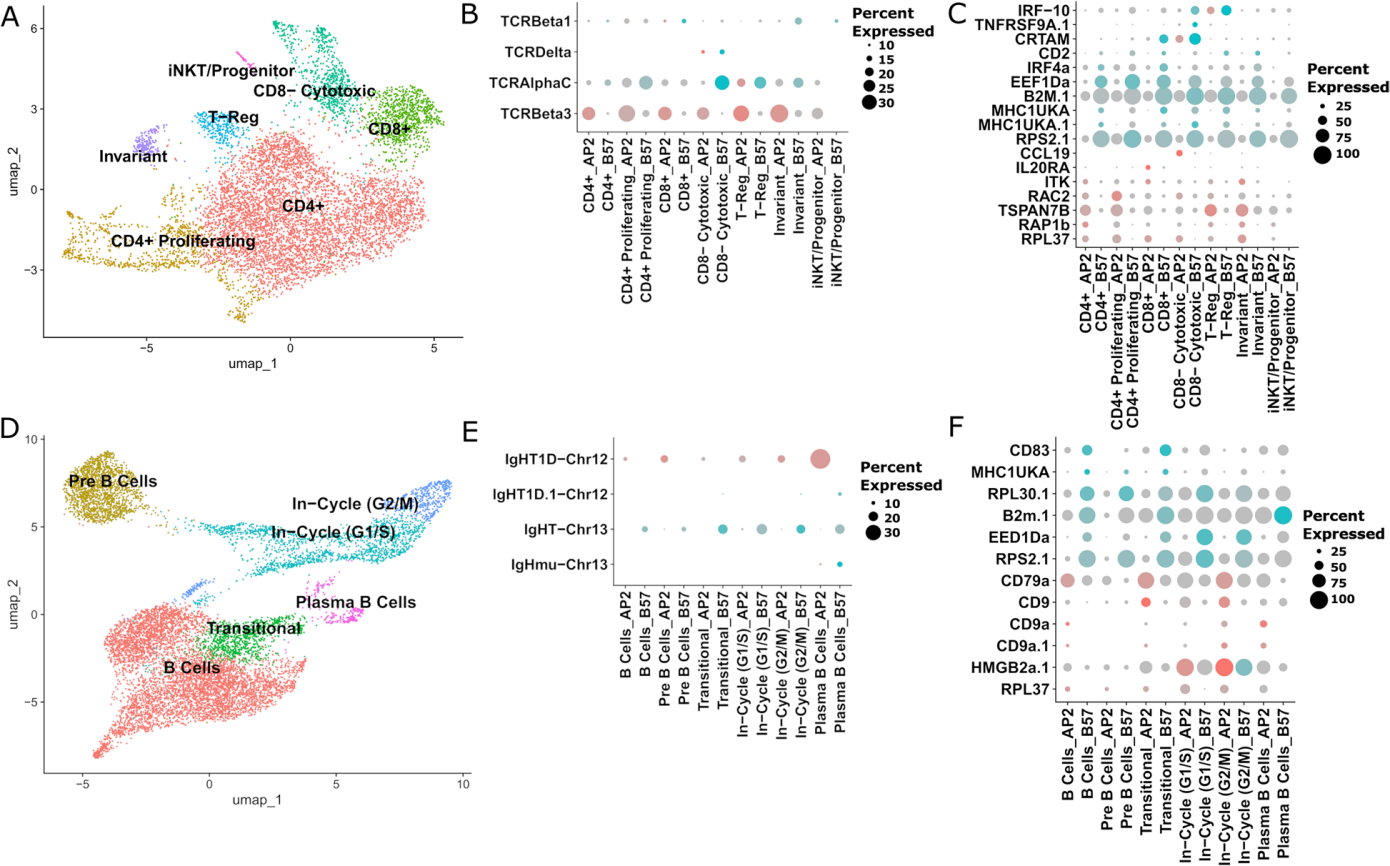
scRNA-seq annotation of rainbow trout head kidney B and T lymphocytes at steady state. **A** UMAP of T-cell populations represented by different colours. **B** Dot plot of expression of T-cell receptor isotypes based on head kidney scRNA-seq data. The size of the dots corresponds to the percentage of cells in a cluster expressing each marker indicated by the legend on the right, while colour intensity represents level of expression in AP2 (red) and B57 (blue) scaled by gene and grey colour represents lower expression. **C** Dot plot of top differential genes identified between T-cell populations of AP2 and B57. **D** UMAP of B-cell populations represented by different colours. **E** Dot plot of expression of immunoglobulin heavy chain isotypes based on head kidney scRNA-seq data. **F** Dot plot of top differential genes identified between B-cell populations of AP2 and B57

Seven T-cell subpopulations were identified and found in similar proportions between AP2 and B57 (Fig. 3A, Additional File 4e). Strikingly, all T-cell subclusters expressed T-cell receptor (TCR) *α*/*β* at some level, with a dominant expression of genes encoding TCRβ1 over the TCRβ2 subtype [53]. We failed to detect expression of the gene coding TCRγ, and while the TCRδ constant gene was detected in a small fraction of CD4^−^CD8^−^ cytotoxic cells, we could not associate this with rearranged functional TCRδ mRNAs or with typical markers of *γδ* T cells (Fig. 3B). The lack of *sox13* expression in any subset further indicated an absence of *γδ* T cells in B57 and AP2 [53]. Within *αβ* T cells, the mutually exclusive expression of *cd4* and *cd8* genes distinguished two main sublineages (Fig. 3A).

To compare AP2 and B57 T-cell transcriptomes, we first performed a pseudobulk analysis of all T cells, which pointed mainly to genes identified in the global pseudobulk analysis (compare Fig. 3C and Fig. 2D). We then applied pairwise differential analyses across AP2 and B57 T-cell subclusters, which revealed a more activated phenotype for cytotoxic B57 subsets in comparison to AP2 (based on higher expression of the markers *cd2*, *irf4*, *crtam*, *tnfrsf9a*, and *irf10* both in CD8 − and CD8 + cytotoxic subsets) (Fig. 3C, Additional File 4f). The expression of *irf10* was also higher in the subset identified as T-regulatory cells (Tregs) in B57. In contrast, AP2 CD8^+^ T cells expressed markers suggesting differences in inflammatory responses and possibly chemotaxis such as interleukin-2-inducible T-cell kinase gene (*itk*), *il20ra*, and the chemokine *ccl19*.

### B cells

Six subpopulations of B cells were identified and annotated by various markers (Fig. 3D). B cells, plasma B cells, and in-cycle (i.e. cell cycle/proliferating) B-cell subsets were annotated in similar proportions in AP2 and B57 fish (Additional File 4b). B-cell precursors (“pre-B”) were proportionally more frequent in B57 (20.2%, 935 cells) than AP2 (11.8%, 723 cells), while transitional B cells were more abundant in AP2 (11.9%, 729 cells) than B57 (5.6%, 260 cells). Building on our recent study of rainbow trout IgH loci [54], we annotated Ig heavy chain isotypes and investigated their expression (Fig. 3E). Both AP2 and B57 plasma cells expressed the highest levels of IgM; strikingly, AP2 expressed IgD, while B57 expressed IgT. In addition to genes related to antigen presentation, such as the two *mhc1uka* paralogs, *b2m.1*, and others found across cell types, *cd83* was highly expressed in naïve and transitional B57 B cells, but not in AP2 (Fig. 3F, Additional File 4f). CD83 regulates MHC II and stabilises its surface expression [55] and may therefore contribute to differences in AP2 and B57 Ag presentation pathways.

### Monocytic cells

**Fig. 4:**
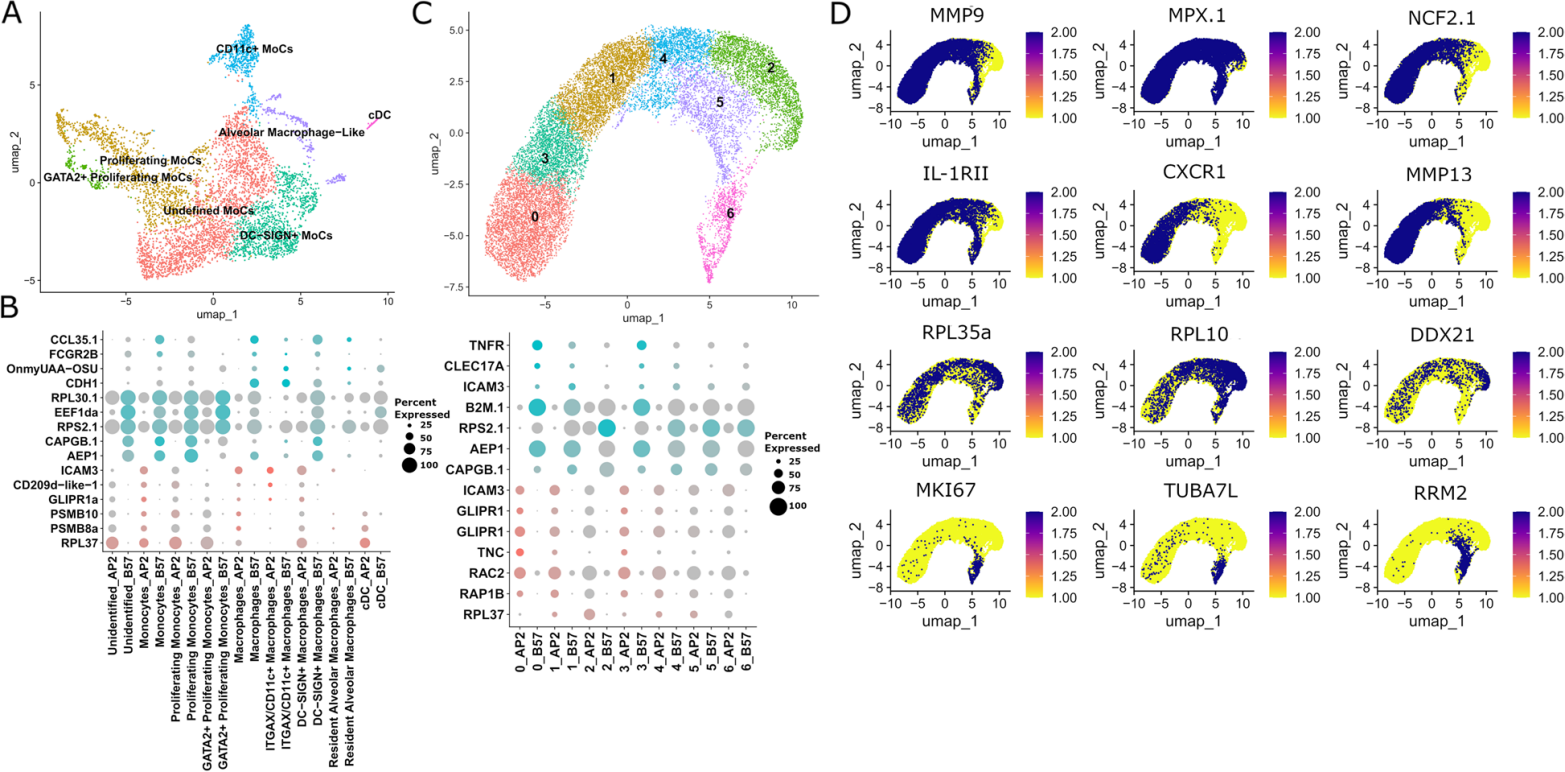
scRNA-seq annotation of rainbow trout head kidney myeloid lineages at steady state. **A** UMAP of monocyte lineage, with subpopulations represented by different colours. **B** Dot plot of top differential markers identified between AP2 and B57. Size of the dots correspond to the percentage of cells in a cluster expressing each marker indicated by the legend on the right, while colour intensity represents level of expression in AP2 (red) and B57 (blue) scaled by gene and grey colour represents lower expression. **C** UMAP of granulocyte subpopulations represented by different colours. **D** Expression of marker genes overlaid on top of the granulocyte UMAP, darker colours represent higher expression while yellow represents low or no expression. The four rows numbered 1–4 represent the following: (1) Marker genes for neutrophils, (2) immune gene expression indicating mature neutrophils, (3) ribosomal protein genes and related interacting gene *ddx21*, and (4) cell cycling genes and tubulin. **E** Dot plot of top differential genes identified between granulocyte populations of AP2 and B57

Re-clustering of the myeloid cells excluding the granulocytes identified nine clusters (Fig. 4A) corresponding to the following mononuclear phagocytes (MNPs) populations [56]: conventional dendritic cells (cDC), alveolar macrophage-like, DC-SIGN^+^ monocytic cells (MoCs), CD11c^+^ monocytic cells (MoCs), proliferating monocytic cells (MoCs 1 and 2), and a last set of undefined monocytic cells (unMoCs). Overall, B57 had a higher proportion of head kidney monocytes compared to AP2 (~ 16%/vs 8%) (Table 1). Among subsets, the unMoC category represented ~ 41% in B57 while only 34% in AP2 (Additional File 4b). The other subsets consistently represented smaller fractions in AP2 than in B57. Pairwise differential analyses identified no major difference between immune pathways expressed in AP2 and B57 subsets. Genes detected specifically in B57 included the chemokine *ccl35.1* and *mhc class I uaa*, while two genes from the 20S proteasome subunit beta family (*psmb8a* and *psmb10*) were highly expressed in several clusters of AP2, but not in B57 cells (Fig. 4B, Additional File 4f), as well as *cd209-d*, *icam3*, and glioma pathogenesis-related 1 (*glipr1*), all involved in MoC biology [56, 57].

### Granulocytes

Clustering of granulocytes produced seven clusters, which appeared difficult to annotate individually (Fig. 4C). The whole granulocyte population expressed the neutrophil markers *ncf*, *mmp9*, and *mpx* (Fig. 4D) and may consist entirely of neutrophils [16]. Despite the lack of cell-specific markers, spatial clustering and expression patterns of genes on the UMAP could indicate several transitional states, reminiscent of the developmental trajectory of Atlantic salmon (*Salmo salar*) granulocytes reported in [16]: *mmp13*, *il-1rii*, and *cxcr1* expression in clusters 0 and 3 points to mature neutrophils, clusters 2 and 4 showed a higher abundance of ribosomal proteins and related interacting genes such as *ddx21* and *pno1*, while clusters 5 and 6 expressed higher levels of cell cycle genes. Due to the lack of defined subsets, we focused on the pseudobulk comparison between AP2 and B57, which identified mainly differential genes common to all cell types. Of note, the receptor of the proinflammatory cytokine TNF (*tnfr*) and the immune lectin *clec17a* were specific to B57 granulocytes (Fig. 4E).

### Erythroid cells

Although Percoll gradient selection of head kidney cells leads to depletion of a large proportion of red blood cells, we recovered a significant population of erythroid cells characterised by high levels of haemoglobin genes (Additional File 5; Additional File 4a). We analysed these cells in the same way as our other leukocyte cell types by re-subsetting and clustering. Within our erythroid population, we identified six distinct subpopulations: erythroid cells formed the largest cluster and were named due to their lack of specific expression and the presence of the largest proportion of haemoglobin genes. Three populations could be described as cell-cycling clusters: G1/S, G2/M, and intermediate by co-expression of both differentiation stages’ markers. Interestingly, two clusters of erythroid cells (named immune I and II) expressed immunity relevant genes. These clusters were characterised by their expression of the ISGs *isg15* and *vig1/rsad2*, and of a homolog of the mammalian thymus-specific serine protease prss16, which is critical for the differentiation of antitumoural CD4 + T cells. Interestingly, comparison between AP2 and B57 immune I and II clusters revealed that the haemoglobin gene (*hbe1*), the aquaporin *aqp1a.1*, the ATP-dependent RNA helicase *dhx37*, and paralogs of the lysozyme *lyg* were more expressed in AP2, while two *rpl* genes, the ubiquitin like protein (*ublp*) and the ISG *parp12* and *vig1/viperin*, were more expressed in B57 (Additional File 5; Additional File 4f).

### RNA-seq revealed divergent responses in AP2 and B57 after VHSV infection

**Fig. 5:**
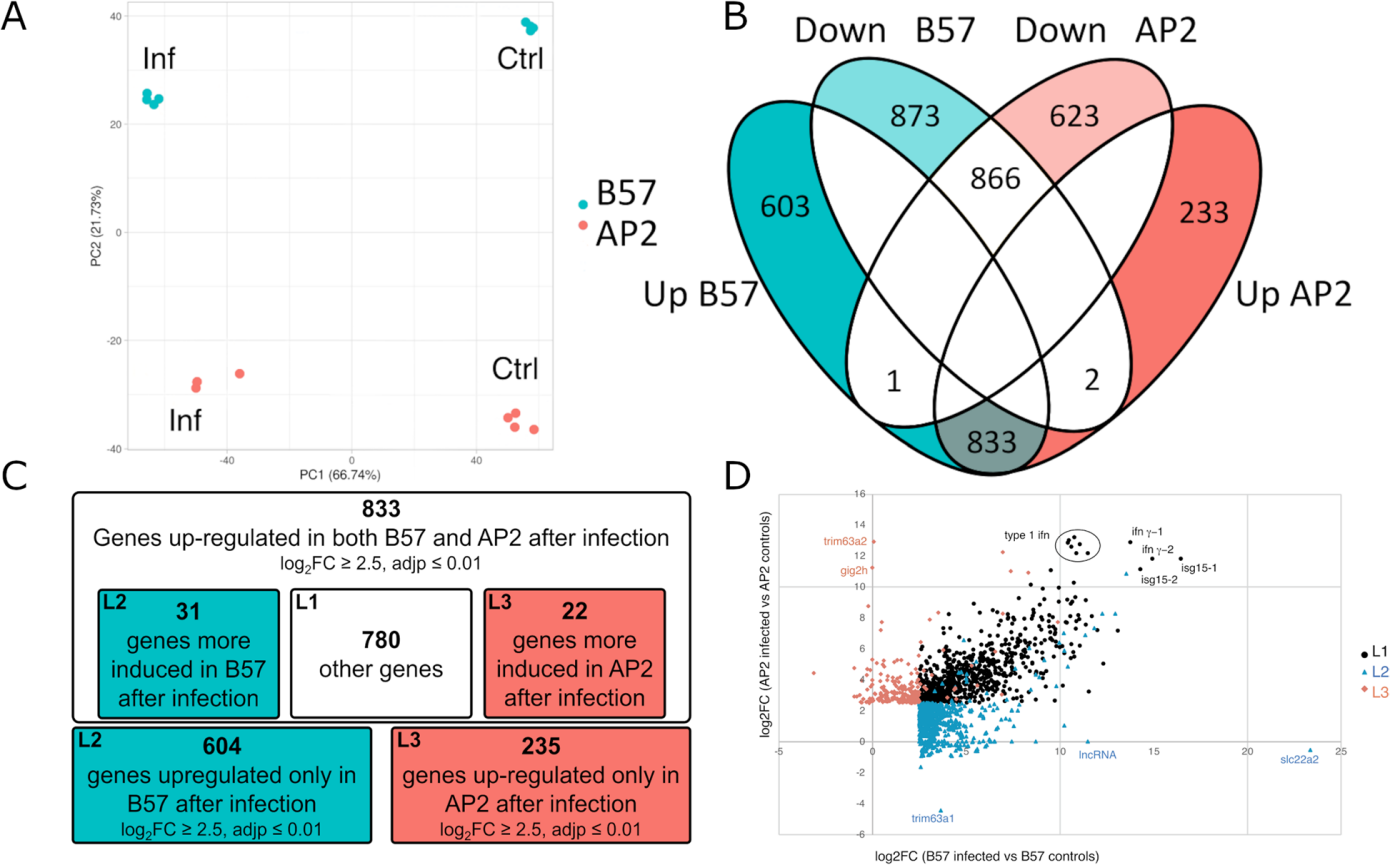
Transcriptome analysis of B57 and AP2 head kidney after infection with VHSV. **A** Principal component analysis of RNA-seq data from head kidney of control or VHSV infected B57 and AP2 fish. Projection on the two first axis is shown (dimension 1: horizontal axis; dimension 2: vertical axis). **B** Venn diagram showing the number of genes significantly up- or down- regulated (|log2FC|> = 2.5, adjp < = 0.01) by VHSV infection based on intra-line comparisons of control and infected fish. **C** Focus on up-regulated genes (either line-specific or common to both lines). L2, genes expressed exclusively in B57 (log2FC > = 2.5, adjp < = 0.01 in intra-line comparison of control and infected fish) or more induced in B57 (log2FC > = 2.5, adjp < = 0.01 in inter-line comparison of B57-infected and AP2-infected fish, inside upregulated genes in both lines in **C**); L3, genes expressed exclusively in AP2 (log2FC > = 2.5, adjp < = 0.01 in intra-line comparison of control and infected fish) or more induced in AP2 (log2FC < = − 2.5, adjp < = 0.01 in inter-line comparison of B57-infected and AP2-infected fish, inside set of genes upregulated in both lines in **C**); L1, remaining genes in the set of upregulated genes in both lines. **D** FC/FC representation of upregulated genes belonging to lists L1 (black dots), L2 (blue dots), and L3 (red dots) defined in C

Head kidney transcriptome responses of VHSV-susceptible AP2 and resistant B57 fish to VHSV infection were analysed in comparison to noninfected controls using RNA-seq. This analysis was performed 3 days after virus injection in order to characterise a fully developed response before the onset of clinical disease and strong physiological stress due to extended lesions. Individual samples were classified by principal component analysis (PCA) into groups reflecting infection status (PC1) and genetic background (PC2) (Fig. 5A). A large number of genes were significantly up- or down-regulated between head kidney of controls and infected fish (9464 genes in B57 and 7846 in AP2 for an *adjp* = < 0.01 and Log2FC > 1 or < − 1; Additional File 6). As the virus induced shut-off leads to a global downregulation of gene expression [58], we focused on genes induced by the infection. A large fraction of VHSV highly induced genes (log2FC > = 2.5 and *adjp* = < 0.01) were up-regulated in both fish lines (Fig. 5B: 833 genes). Fewer genes were induced only in 1 fish line (603 genes only in B57 and 233 genes only in AP2; Additional File 6), and very few genes were upregulated in a line while down-regulated in the other (*trim63a* in B57 and 2 genes in AP2, an Igκ constant gene and an uncharacterized gene (Fig. 5B and Additional File 6). The same trend was observed with a threshold log2FC > = 1 and *adjp* = < 0.01 (Additional File 7).

We also quantified the expression of the VHSV glycoprotein (G) gene in head kidney and spleen of all fish using qPCR. We detected the G mRNA in all samples from infected fish, but not from controls. There was no significant difference in expression level between AP2 and B57 head kidney samples (Additional File 8).

### The B57-specific transcriptome response to VHSV is stronger than in AP2 and is enriched in proinflammatory genes

Among the 833 genes highly induced in both AP2 and B57 head kidney, we distinguished genes upregulated with comparable fold change in AP2 and B57 (with |Log2FC [$\frac{infected AP2}{infected B57}$]|= < 2.5, *adjp* < = 0.01, list L1) and genes induced more in AP2 (Log2FC [$\frac{infected AP2}{infected B57}$] > = 2.5, *adjp* < = 0.01) or more in B57 Log2FC [$\frac{infected B57}{Infected AP2}$] > = 2.5, *adjp* < = 0.01) (Fig. 5C). Genes highly induced only in AP2 (235) or B57 (604) were selected with Log2FC [$\frac{Infected }{Control}$]|> = 2.5 and *adjp* < = 0.01.

The common response (list L1) involved 780 genes, the B57-specific response (list L2) 635 (604 + 31), and the AP2 specific response (list L3) only 257 (235 + 22), showing how different the head kidney transcriptome response induced by the virus was between the two fish lines (Fig. 5D).

The importance of type I and type II IFN upregulation in both AP2 and B57 is illustrated by the LogFC/LogFC graph in Fig. 5D. *isg15* appears as one of the most upregulated ISGs, as observed in [59]. We previously described the repertoire of ISG conserved in salmonids, zebrafish, and human, in contrast to genes without orthologs or with orthologs that are not responsive to IFN in these species [15]. To explore the evolutionary features of genes involved in the response of AP2 and B57 fish, we classified genes of L1–L3 into conserved ISGs, non-conserved ISGs, and genes induced by viral infection, but not by poly I:C (a strong inducer of type I IFN responses) or recombinant type I IFN.

Genes induced by VHSV both in AP2 and B57 (L1) comprised a large part of the core conserved ISG (as defined in [15]) which were highly induced in our datasets (136 out of 177) and 484 other non-conserved ISGs (Table 2). Only 20% of L1 genes (160 genes, Table 2) are not classified as ISG in our evolutionary analysis [15]. In contrast, the B57 (or AP2)-specific response (L2 and L3) comprised very few conserved ISG (about 5%), about 45–50% of non-conserved ISGs, and 45–50% of non ISGs, based on the classification reported in [15]. The B57-specific response comprised 27 conserved ISGs, including four *samd9*-like genes potentially involved in translation regulation through tRNA-Phe targeting, the transcription factor *irf7* that is involved in type I IFN induction, and several key factors of the *jak/stat* pathway (*irf9*, *stat2*, *nmi/ifi35*, *dtx3)* (Additional File 6). The other ISGs (235 genes) comprised many TRIM genes from subsets typically involved in antiviral responses such as fintrims (teleost-specific TRIM genes) or BTRS (bloodthirsty-like trims) [60] and nucleic acid sensors like *tlr13* and *tlr8b*. The last category gathered genes upregulated by VHSV infection, but not induced by poly I:C or type I IFN (373 genes). These genes comprised a number of key pro-inflammatory genes like *irak1* and the key cytokines *il1b*, *tnfa*, *tnfb*, and *cxcl8*. In this last category, a few antiviral genes (two type I IFN genes and the cholesterol hydroxylase *ch25h*) were also upregulated, along with several members of the TNF superfamily (*tnfsf1b*, *tnfsf10a*, *tnfsf25*).

**Table 2:** Genes upregulated AP2 and B57 head kidney or specifically/mainly in each rainbow trout line (lists L1–3)

	L1 Upregulated in AP2 & B57 **780 genes** (80% ISG)	L2 Upregulated or more induced in B57 **635 genes** (40% ISG)	L3 Upregulated or more induced in AP2 **257 genes** (51% ISG)
Conserved ISG	136 genes Response to virus (GO:0009615) Regulation of immune effector process (GO:0002697)	27 genes Response to bacterium (GO:0009617) Innate immune response (GO:0045087)	14 genes Negative regulation of viral process (GO:0048525) Antigen processing and presentation (GO:0019882)
Nonconserved ISG	484 genes Innate immune response (GO:0045087) Cytokine-mediated signalling pathway (GO:0019221)	235 genes Negative regulation of cytokine production (GO:0001818) Positive regulation of cytokine production (GO:0001819)	116 genes Innate immune response (GO:0045087) External encapsulating structure organisation (GO:0045229)
VHSV induced but not polyI:C induced	160 genes Cytokine activity (GO:0005125) Positive regulation of cytokine production (GO:0001819)	373 genes Positive regulation of cell development (GO:0003720) Cytokine activity (GO:0005125)	127 genes Natural killer cell activation (GO:0030101) Adhesion of symbiont to host cell (GO:0044650)

The AP2-specific response comprised 14 conserved ISGs, including the transcription factor *stat1a*, the antiviral viperin *(rsad2)*, *tap*2, and *tapasin* that are each involved in antigen processing and presentation (Additional File 6). Among non-conserved ISGs (116 genes), specific gene groups comprised transcription regulators *gigh* (four genes) and antiviral GTPases *gimap-like* genes (6 genes). Only four *fintrim* genes were classified in this group, in contrast to B57. Table 2 also shows the main terms identified by a gene ontology analysis by Metascape (see also Additional File 9).

Transcriptome response to VHSV infection involves a number of multigene families (Additional File 10). Within these families, different members were often induced either in AP2 or in B57. Such alternative induction of different genes in AP2 and B57 may represent haplotype-specific specialisations involved in resistance or susceptibility mechanisms. For example, the large set of non-conserved ISGs induced either in AP2 or B57, comprised many *trim* genes, in particular *fintrim (ftr)*. The large set of induced *gimap* genes were in the majority induced in both AP2 and B57, but a few members were line specific. In contrast, most members of the *samd9* family were AP2- or B57-specific. Such induced gene families also include many cytokines/chemokines subsets, including type I IFN, IL10, IL17, and TNF families, which have complex phylogenetic relationships with their mammalian homologs. For example, a surprising diversity of type I IFN genes (23 genes) was induced in AP2 and/or B57 (Additional File 10).

### scRNA-seq reveals remarkable contrast in immune cell types responsive to VHSV in AP2 and B57

We next compared the differentially expressed genes identified from bulk RNA-seq analysis of AP2 and B57 head kidney tissue of infected fish with the corresponding single-cell data (Additional File 11). In contrast to the RNA-seq/scRNA-seq comparison of control samples, upregulated genes detected in the bulk analysis fit only partially to the scRNA-seq results combining all cells, although general trends were retained (Additional File 11). Critically, cells removed by the density gradient preparation included a large proportion of red blood cells, as well as endothelial, epithelial, and stromal cells. These cells can display a strong response when infected [61–63], which is integrated in the whole tissue analysis, but not reflected in our single-cell data.

**Fig. 6:**
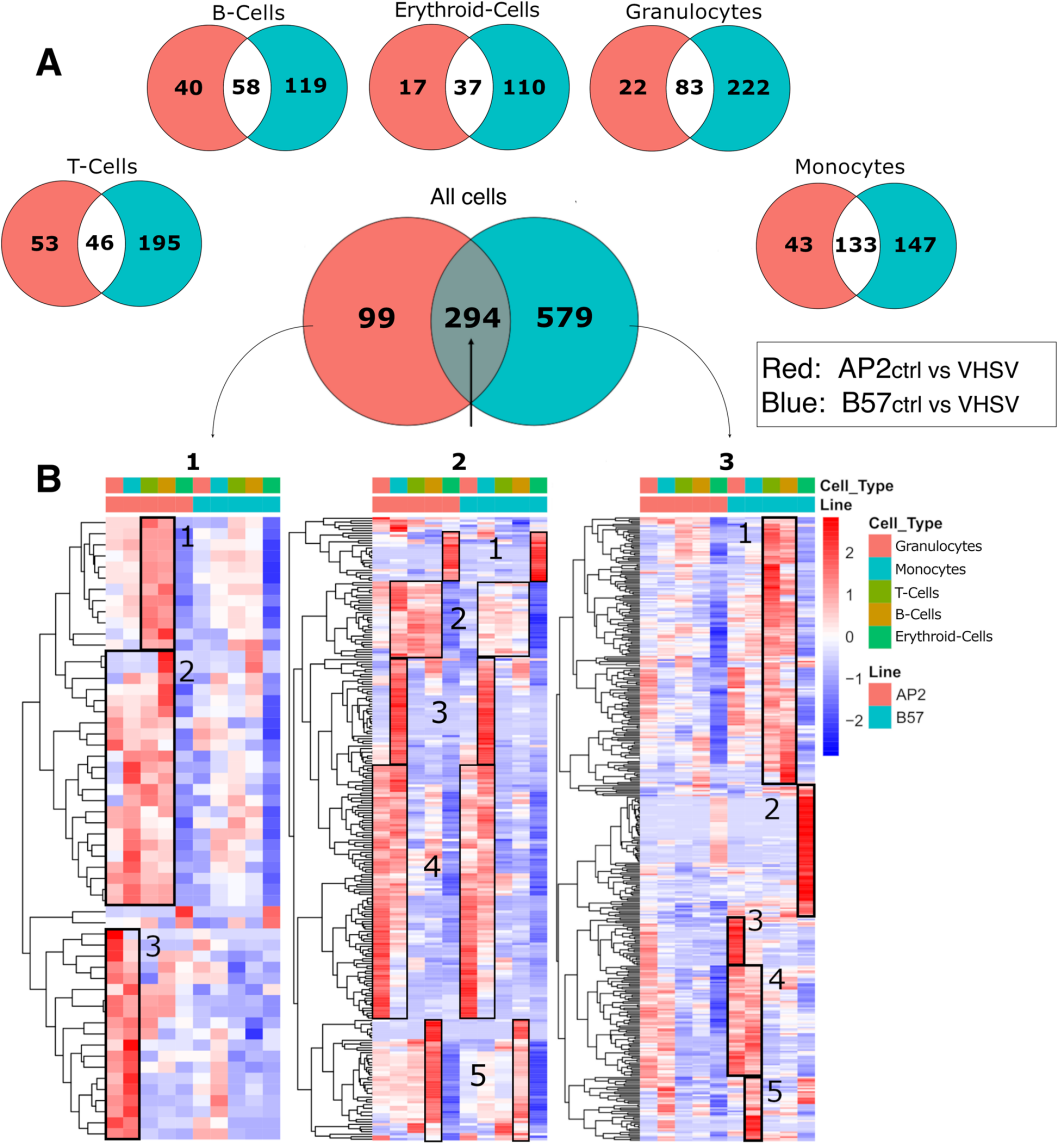
scRNA-seq analysis of head kidney immune cells from VHSV-infected AP2 and B57 fish. **A** Venn diagrams of significantly upregulated genes identified by pairwise analysis of each cell type between VHSV-infected and control samples of head kidney leukocytes, with AP2 in red and B57 in blue. **B** Heatmaps of **A** integrated nonredundant Venn diagram, left (AP2), middle (shared), and right (B57). Numbered boxes were used to highlight gene sets with particular expression patterns across cell types; gene names can be found in Additional File 12

To gain insights into the leukocyte subsets responsible for the response to VHSV, differential expression analyses were performed using scRNA-data per cell type from infected and control fish. Comparisons were performed for granulocytes, monocytic cells, B cells, T cells, and erythroid cells, and the results were integrated to produce a global differential analysis between AP2 and B57. The same trend was found as previously observed in the RNA-seq analysis, with a lower number of genes specifically induced in AP2 (99) compared to those specifically induced in B57 (579) following VHSV infection; the shared response comprised 294 genes (Fig. 6A; Additional File 12).

The expression of these shared response genes was distributed across cell types and near identical between AP2 and B57 (see Fig. 6B1-3). A first gene set (Set1 in Fig. 6B2) induced in the erythroid lineage comprised genes with roles in heme metabolism (*alad*, *blvrb*), iron metabolism and ferroptosis (transferrin receptor *tfrc*, ferritin *fth*), and several genes (peroxydases *prdx1*, *prxl2a*, *tspo*) regulating the oxidative burst. A second set (Set2 in Fig. 6B2) comprised genes induced by VHSV in monocytic cells, B and T lymphocytes, encoding ribosomal proteins (12 genes), and ISGs (*dhx58*, *2 helz2*, *4 rnf213*, *galectins*, *a parp and a gig2-like*, *mx1*, *ifi44l*, and several *trim* genes), and a third set (Set3 in Fig. 6B2) was specific to monocytic cells, including extracellular matrix or lysosomal genes (including two granulin genes, *timp2*, *psap*, *ifi30*), a cathepsin, and several chemokines. The largest cluster (Set4 in Fig. 6B2) was induced in granulocytes and monocytic cells, including key genes involved in inflammation: *il1b*, *mmp9*, two lysozymes, the myeloperoxydase *mpo*, five cytochrome oxydases, *ncf1* (a subunit of neutrophil NADPH oxidase involved in superoxide production), *aloxap* (an arachidonate 5-lipoxygenase activating protein), the activation transcription factor *atf3*, several tumour necrosis factor (TNF)-induced genes (*tnfaip-2* and *−3* like), and a thioredoxin gene. Finally, a last cluster (Set5 in Fig. 6B2) contained genes induced only in B cells, mainly immunoglobulin light chain genes and IgD heavy chain likely associated with B-cell activation, ribosomal proteins, death-associated protein-like 1, and rRNA genes, suggesting changes in protein synthesis perhaps connected to B-cell differentiation into antibody-secreting cells. Interestingly, Set5 also comprises the ISG *ifi44l* and the transcription factor *irf1*, which promotes T-cell-independent B-cell responses [64].

In contrast, genes specifically upregulated in AP2 or in B57 showed divergent cell type distributions (Fig. 6 B1 and 3, respectively). Genes induced mainly/only in AP2 (Fig. 6B1) were classified in three main sets (Set1–Set3):

- Set1 genes (Fig. 6B1), induced in B and T cells, encoded mainly ribosomal proteins, pointing to a change of translation activity in these cells.
- Set2 genes (Fig. 6B1) were overall upregulated in leukocytes but not in the erythroid lineage. These genes showed a clear ISG signature, in accordance with our RNA-seq analysis (*galectin9*, *ifi44l*, *rnf213*, *dhx58*, *helz2*, and an *mx* gene).
- Set3 genes (Fig. 6B1) were mainly induced in AP2 (but not B57) granulocytes and monocytic cells and comprised many other typical ISGs (*rsad2*, two *isg15* genes, another *aste2*, *ifit5*, *ifi27*), underscoring the central importance of the type I IFN response in AP2-specific response both in lymphocytes and myeloid cells.

Genes induced specifically in B57 (Fig. 6B3) showed a distinctive expression pattern with five main clusters Set1–Set5: a large cluster of genes induced in B57 only and mainly in B and T cells (Set1 in Fig. 6B3) revealed a stronger response compared to AP2 lymphocytes. Set1 comprised genes encoding ribosomal proteins and translation initiation factors (*rpl3*, *rplp0*, *rpl7*, *rps13*, *eif3ha*, *eif3m*, *eif1*, *eif3g*, *eef2b*, *eif3eb*, *eif3s6ip*), suggesting an upregulation in protein synthesis as observed in AP2, but through different genes. The Set1 lymphocyte cluster also contained key ISG (*irf3*, *stat1a*, *calcoco2*, a *cd9 paralog*, *irf1b*, *ifi35*) and 15 trim genes (from the virus-induced *fintrim* (*ftr*) and bloodthirsty-like trim (*btr*) families). Transcription factors (*batf*, *atf4b*, *ikzf1*, *irf4a*, *stat4*, *pax5*, *btf3*), several components of the NF-kB pathway (two *nfkbiaa* genes, *nfkb2*), and the kinase *map3k14* suggested a specific modulation of cell activation. Other important responses were the induction of cytokine receptors *il2rb* and *il7r*, which were not observed in AP2 lymphocytes. Set2 genes (Fig. 6B3) were upregulated only in cells from the B57 erythroid lineage and including genes involved in erythroid differentiation (*epor*, *gata1*, *rhag*, *ank1*) and iron metabolism (*fthl*, *hbz*, *frrs1*, *tfrc*, *rfesd*, *h2az2*). Set3, Set4, and Set5 were more induced in B57 but showed a similar trend in AP2. Set3 genes (Fig. 6B3) were only upregulated in granulocytes and comprised important inflammatory genes such as *il1b*, *arg2*, *tnfaip3*, *mmp9*, *13* and *25*, and *nlrc3l*. Set4 genes (Fig. 6B3) were induced both in B57 granulocytes and monocytic cells, comprising cell type markers such as *lyz* and *csf3r*, three ATP-synthase genes, the cathepsin *ctss*, the oxidative stress response transcription factor *nfe2l2*, *cd40*, *irf8-2* [30], and several proteasome components. The last cluster was induced in monocytic cells only and comprised rather similar genes to Cl4 with two ATP synthase genes, *irf8.1* from chr26 [30] as well as cathepsins *ctsB*, *ctsC*, and *ctsZ*.

## Discussion

The clinical variability between individuals in the course of infection by a pathogen is remarkably high, from asymptomatic to fatal diseases, as illustrated by the recent pandemic of COVID-19 in human populations [65]. In this work, we analysed transcriptome response to VHSV of the head kidney from the resistant line B57 and from our most susceptible rainbow trout line, AP2. Combined bulk RNA-seq and scRNA-seq revealed highly contrasted transcriptome responses to VHSV infection, involving distinct gene modules in different leukocyte populations between the resistant and susceptible genotypes of fish. This study offers a comprehensive framework for exploring the evolution of antiviral resistance in vertebrates.

### AP2 and B57 head kidney transcriptomes at steady state do not provide an obvious explanation for differences in resistance to VHSV

Our initial analysis of VHSV resistance aimed to identify potential phenotypic differences in cell composition and expression profiles between head kidney from resistant and susceptible fish at steady state. We originally hypothesised that resistance might stem from potential differences in cell developmental pathways or an enhanced expression of antiviral genes. Overall, genes with a contrasted expression between AP2 and B57 head kidney at steady state represented a relatively small fraction (about 10%) of the gene repertoire expressed in this tissue. Unexpectedly, more conserved ISGs were expressed at higher levels in the susceptible AP2 than in the resistant B57 line, including antiviral genes such as *mx1*, *ifitm3*, *ifit5*, and *rsad2*. However, the majority (70%) did not display significant difference of expression. Based on scRNA-seq, the cellular composition of head kidney leukocytes and erythroid cells appeared largely identical between the two isogenic rainbow trout lines. Only the monocytes displayed a notable difference with an increased proportion in AP2. Strikingly, we found within the erythroid lineage two small cell clusters expressing a number of ISG such as *isg15* and *rsad2/viperin*. It has been proposed previously that fish red blood cells, which are nucleated, may express immune genes and play a role in innate immunity [66, 67]. We show here that while erythrocytes were mostly excluded from our scRNA-seq analysis, some erythroid cell subsets express several ISGs, with contrasted expression levels in AP2 and B57; for example, *isg15* and *rsad2* were more expressed in B57.

Our single-cell analysis also revealed a higher expression of several key genes of the MHC class I pathway (*b2m.1*, *OnmyUAA-OSU*) across all major lineages in B57, compared to AP2, suggesting that B57 is better equipped to induce antiviral cellular responses. Strikingly, the differences between AP2 and B57 transcriptomes were very consistent, whether assessed in the whole head kidney tissue by global RNA-seq or in density gradient-purified cells by scRNA-seq. Thus, the absence of cell populations removed by density gradient separation had little contribution to the transcriptomic divergence between AP2 and B57 at steady state, indicating that this divergence primarily arose within leukocytes. Overall, our analyses in uninfected fish offered no obvious explanation for the resistant and susceptible status of B57 and AP2 fish, except maybe a higher expression of MHC class I genes in B57.

### AP2- and B57-specific responses are enriched in non ISGs and reveal different reactions of leukocyte subsets

Many more genes were highly induced by VHSV specifically in B57 than AP2, with a majority of genes induced in both lines. This was in stark contrast to similar studies comparing resistant and susceptible viral disease phenotypes in Atlantic salmon (IPNV) and common carp (cyprinus herpes virus type 3: CyHV3). Upon infection, Atlantic salmon fry susceptible to IPNV exhibited a more extensive transcriptome response than resistant fish [68]. Similarly, a stronger response was observed in the spleen of common carp (*Cyprinus carpio*) susceptible to CyHV3 compared to resistant fish [69]. In our data, both AP2 and B57 responses comprised a common type I IFN response, besides a pro-inflammatory gene set specific to B57 and a group of type I IFN pathway genes specific to AP2. This was in contrast to our previous observations on fibroblast cell lines, in which B57 showed a stronger and faster type I IFN response than in the fibroblastic line A22 derived from another susceptible background [10, 13]. Altogether, these observations highlight the diversity of transcriptome responses of resistant and susceptible genetic backgrounds to various viruses; differences of response are often complicated by composition and kinetics between tissues [10, 13]. Thus, we utilised scRNA-seq to provide further insights into respective response contributions of head kidney leukocytes. Remarkably, the AP2- and B57-specific arms of the response were expressed by different cell types: the former predominantly in monocytic cells and granulocytes while the latter was more specific to B and T lymphocytes, with an additional module in erythroid cells. In addition, several class I pathway genes were more expressed across leukocyte subsets in B57 than in AP2, reflecting divergent orientations of T-cell immunity. These contrasted immune responses likely contribute to differences in susceptibility to pathogens. In keeping with this, AP2 fish are susceptible to VHSV and infectious hematopoietic necrosis virus (IHNV) but resistant to *Flavobacterium psychrophilum*, whereas B57 shows the opposite pattern, being resistant to VHSV and IHNV while susceptible to *F. psychrophilum* [14, 70].

### The repertoire of genes involved in both AP2 and B57 responses to VHSV is enriched in evolutionary conserved interferon-stimulated genes

A large fraction of the transcriptome response induced by VHSV in the rainbow trout head kidney was common to AP2 and B57 lines. This was in contrast with the very little overlap between the gene sets induced in the spleen of common carp resistant and susceptible to CyHV3 [69]. Strikingly, this shared response comprised most of the evolutionary conserved ISGs, which we have previously identified [13, 15, 60, 71]. Conversely, genes induced directly by the viral infection were proportionally modest compared to the type I IFN-dependent response, within genes induced in both AP2 and B57. This highlighted that the type I IFN pathway appears as a more conserved module that was overall present across the rainbow trout population from which the doubled haploid lines were derived. Interestingly, in many multigenic ISG families, a large proportion of the members responding to VHSV were part of the shared response, perhaps reflecting their complementary functions or shared regulatory mechanisms. When investigating the common response in the scRNA-seq data, we observed that cell types in B57 and AP2 expressed VHSV-induced genes in a remarkably similar manner, underlining the conservation of the response in susceptible and resistant fish. This well-developed common response largely based on type I IFN therefore does not explain the fish resistance status. It is interesting to note that VHSV induces an early and fast response in B57 at least in fibroblastic cells, which certainly contributes to its lower susceptibility [13].

## Conclusions

Our work presents a first detailed annotation of the transcriptomic response to viral infection in immune cells from genetically resistant and susceptible fish. It highlights the divergent roles of lymphocytic and monocytic cells and challenges the prevailing view that inflammatory responses are predominantly detrimental (Table 3). It offers both a comprehensive basis for evolutionary studies of the role of different immune cell subsets in antiviral response and resistance and a frame for future analysis of the response in resistant and susceptible backgrounds. However, our data are restricted to head kidney leukocytes from adult fish. Future studies will extend our study to other cell types and tissues, for example skin at the VHSV portal of entry [4]. It will also be interesting to compare transcriptome differences during development and maturation of the immune system.

**Table 3:** Overview of the main conclusions of this study

	**AP2 specific** **(VHSV susceptible)**	**Observed both in** **AP2 and B57**	**B57 specific** **(VHSV resistant)**
**At steady state**	High expression level of a number of core ISG	Overall similar frequency of main leukocyte types	High expression Ag presentation genes (*mhc I*, *β2m*…)
**After VHSV infection**	Genes mainly/only induced in AP2 with specific expression pattern, with ISG mainly in granulocytes and monocytes	Strong upregulation of many core conserved ISGs in both lines across multiple cell types	Genes mainly/only induced in B57 with specific expression pattern, with ISG and *ftr* mainly in lymphocytes, and many inflammatory genes in granulocytes and monocytes

## Methods

### Animal studies

Immature rainbow trout (~ 153 g) were raised in the freshwater fish facilities of the Institut National de la Recherche en Agriculture et environnement (INRAE, Jouy-en-Josas, France). All fish experiments were carried out in accordance with the recommendations of the European Union guidelines for the handling of laboratory animals (http://ec.europa.eu/environment/chemicals/lab_animals/index_en.htm). The experimental protocols were approved by the INRAE Institutional Ethics Committee “Comethea” (permit license no. 15–60). All fish were healthy and monitored throughout the study. Fish at all times were kept in 300-L tanks supplied with recirculating dechlorinated water with a flow rate of 1000 L/h, temperature of 10 °C, and a photoperiod of 10:14 h (light:dark). A computerised control system was used to monitor water parameters over the duration of the stimulation. Fish were sacrificed by an S1K method through overexposure to benzocaine (100 mg/L) and subsequent destruction of the brain, followed by bleeding of the gills to remove as many erythrocytes as possible from the sampled tissue.

### VHSV infections

Viral infection studies were performed in two separate experiments for the RNA-seq and scRNA-seq experiments.

For the RNA-seq study, initially 16 fish from both AP2 (susceptible) and B57 (resistant) were pit-tagged and then evenly separated into two tanks. Fish were then intraperitoneally (I.P.) injected with either VHSV (virulent strain 07–71 [72], 5 × 10^5^ plaque-forming units (PFU)/fish) or phosphate-buffered saline (PBS) and returned to separate control and infected tanks (*n* = 8 per isogenic line and treatment) for 72 h. Head kidney tissue (100–200 mg) was sampled from each fish and stored in RNA later at 4 °C overnight before long-term storage at − 80 °C.

For the scRNA-seq, experiments were performed with two fish infected with VHSV and with two controls (i.e. noninfected fish) for each rainbow trout line in exactly the same way as the bulk RNA-seq experiment. Further to this, a later repeat experiment with VHSV was then performed on two more B57 fish for further comparison. While immature fish weighing approximately 150 g were too large to be susceptible to VHSV infection, our objective here was to characterise the transcriptomic response of head kidney leukocytes to the virus, rather than to assess susceptibility or monitor fish survival post-infection. Our previous studies have clearly demonstrated that VHSV triggers distinct responses in cells derived from susceptible and resistant isogenic fish [10, 13].

### scRNA-seq experiment and data analysis

Following euthanisation of animals designated for scRNA-Seq, the full head kidney tissue from each individual was aseptically extracted and placed into separate falcon tubes filled with 20 mL of extraction media (L15 [Gibco], 2% FBS, and 0.02% EDTA) and kept on ice. Head kidney leukocytes were purified using a 51% Percoll gradient and then diluted to around 1000 cells/μL. Cell suspensions were loaded into the Chromium Controller (10 × Genomics) using a Chromium Next GEM Single Cell 5′ kit v2 (10 × Genomics) to generate a droplet emulsion. The scRNA-seq libraries were then prepared using the Single Cell 3′ Reagent Kit v3.1 from 10 × Genomics (Pleasanton, CA, USA) following the manufacturer’s protocol, aiming to recover 5000 cells per sample library. Sequencing of scRNA-seq libraries was done using an Illumina NextSeq500 (San Diego, CA, USA) using a High Output kit v2 (150 cycles) kit generating 150 base pair (bp) paired-end reads. Fastq files were analysed using the Cell Ranger software (10 × Genomics, version 5.0.1), including alignment, filtering, and quantitation of reads on the rainbow trout genome for generation of feature-barcode matrices. The rainbow trout reference genome (USDA_OmykA_1.1, GCA_013265735.3) was downloaded from Ensembl (version 110). STARsolo (2.7.10a) [73] was used to map raw read files (fastq) from the scRNA-seq dataset and quantify gene expression per cell. The unfiltered count matrices from each sample following mapping were then loaded into R. Initial quality control filtered cells with fewer than 200 genes or more than 3000, indicative of empty droplets or doublets. The remaining data was then assessed computationally for doublets from package scDblFinder (1.14.0) [74], with only singlet detected cells kept for remaining analysis. Replicates from each isogenic line and treatment group were then integrated to remove sample batch effects using Harmony [75], and downstream analysis was performed using a standard Seurat workflow of the following: NormalizeData (“LogNormalize”), FindVariableFeatures (*n* = 2000), ScaleData (based on expressed genes), RunPCA (based on variable features), RunUMAP (standard seurat parameters bar reduction = “harmony” and dims = 1:24), FindNeighbors (standard seurat parameters bar reduction = “harmony” and dims = 1:24), and FindClusters (resolution = 0.8) [76]. The number of dimensions used for the projections was decided upon by use of elbow plots. Subsequent individual analysis of cell types (B cells, T cells, granulocytes, monocytes, and erythroid cells) was performed by subsetting cells from these lineages and renormalising and clustering according to the steps previously listed with Seurat. The full code used in the scRNA-seq analysis can be viewed in the Additional File 5.

### RNA-seq experiment and analysis

For total RNA extraction, head kidney tissue was homogenised with ceramic beads in a Precellys Evolution tissue homogeniser in 1 mL of TRIzol. Total RNA was then extracted as per the manufacturer instructions for a standard TRIzol RNA extraction using chloroform and precipitated with isopropanol. Concentration and purity of the RNA were estimated using a NanoDrop 2000C Spectrophotometer, alongside further confirmation on an Agilent Bioanalyzer 2100 to generate RNA integrity (RIN) values. Libraries were prepared and sequenced by Novogene using the Illumina NovaSeq 6000 platform to generate around 30 million paired-end 150-bp reads per sample. The nf-core/RNA-seq pipeline (version 3.14) [77] was utilised to analyse raw paired-end reads (150 bp) from each sampled fish (*n* = 16). Raw reads were filtered by removing adapters, poly-N more than 5, and low-quality reads (more than 40% base with *Q* < 15) by fastp. Then, filtered reads were mapped with STAR to the same rainbow trout reference genome used for the single-cell analysis. The following STAR parameters were used to consider gene counts only for reads mapping coherently and unambiguously: TranscriptomeSAM, outFilterMultimapNmax = 1, and twopassMode None. Raw counts for each annotated gene were quantified using Salmon [78], taking into account gcBias, seqBias, and posBias. Differential expression analysis was performed using SARTools (version 1.8.1) [79], with default parameters and an alpha value of 0.01, employing a variance-stabilising transformation (typeTrans = VST). The analysis was conducted pairwise between control groups (AP2 or B57) and infected groups (AP2 or B57), resulting in four lists of comparisons: controls AP2 vs. infected AP2, controls B57 vs. infected B57, controls B57 vs. controls AP2, and infected B57 vs. infected AP2. Finally, gene set enrichment analyses were conducted on the lists of differentially expressed genes using Metascape [80], with a focus on Gene Ontology (GO) biological processes and molecular functions. To maximise the information obtained, the best human blast hit for each trout gene was selected for the analysis, along with a background set of all expressed genes (sum of raw count > = 10 in at least one line). To obtain these best blast hits, the rainbow trout proteome was retrieved and filtered to retain only the longest isoform for each gene. Protein sequences were subjected to blastp using Diamond v2.0.9.147 [81] against the human (GCA_000001405.28) and zebrafish (GCA_000002035.4) peptide sequences retrieved from Ensembl, and all parameters were set to default except setting − max-target-seqs to 1 and − outfmt to 6.

### Quantitative real-time PCR

RNA was extracted as previously described in the section “RNA-seq experiment and analysis”, with the inclusion of spleen tissue. First-strand cDNA was synthesised from 845-ng RNA using a Takara PrimeScript RT Master Mix cDNA kit. First-strand cDNA samples were diluted fivefold (working stock) with RNase/DNase-free water and stored at − 20 °C. Real-time PCR (qPCR) analyses were performed with a Bio-Rad CFX Duet Mastercycler. All assays were carried out in 25-µL reactions on 96-well plates in duplicates. Each reaction mix contained 2 µL of cDNA, 12.5 µL of Takara TB Green Premix Ex Taq, and 10.5 µL of a forward and reverse primer solution, achieving a final concentration of 200 nM for each primer. PCR cycling conditions were 1 cycle of 95 °C for 1 min, followed by 40 cycles of 95 °C for 10 s and then 60 °C for 30 s. Melting curve analysis (thermal gradient from 60 to 95 °C) was then used to confirm the amplification of a single product. Each plate also included “no template” negative controls in duplicate (cDNA replaced with water). Efficiency was calculated for each primer from a serial dilution PCR. Target gene expression for the VHSV-G gene (F — GGGGCCTTCCTTCTACTGGTACTC; R — CGGAATCCCGTAATTTGGAAT) was normalised to *β*-actin (F — GGGAGAAGATGACTCAGATCATG; R — GGTGGTACGGCCAGAGGC), rps29 (F — GGGTCATCAGCAGCTCTATTGG; R — CCAGCTTAACAAAGCCGATGTCG), and elf1α (F — CACTGCTCAAGTAATCATCCTG; R — CACAGCAAAACGACCAAGAG). Relative expression levels were calculated for each tissue and line, comparing infected and noninfected fish.

## Supplementary Information

Additional file 1. Table S1- RNA-Seq differential analysis of AP2 and B57 head kidney transcriptomes at steady state.

Additional file 2. Table S2—Gene ontology analysis of genes with contrasted expression between AP2 and B57 head kidney at steady state.

Additional file 3. Table S3—Comparison of core conserved ISG expression in AP2 and B57 head kidney transcriptomes.

Additional file 4. Table S4—Single cell transcriptomic analysis of head kidney leukocytes.

Additional file 5. Further analysis of single cell data of T cells, B cells, Monocytes, Erythroid cells, HSC-Like, Thrombocytes and NK-Like, and code used for single cell analysis.

Additional file 6. RNA-Seq Differential analysis of AP2 and B57 head kidney transcriptomes from VHSV infected fish.

Additional file 7. Figure S1- RNA-Seq Venn comparison of differentially expressed genes in AP2 and B57 head kidney transcriptomes after infection.

Additional file 8. Figure S2—Quantification of VHSv G mRNA by RT-qPCR from spleen (S) and head-kidney (HK) of infected fish compared to controls.

Additional file 9. Table S6—Gene ontology analysis of conserved ISG, non-conserved ISG and genes induced by VHSV from L1, L2 and L3 lists.

Additional file 10. Table S7—Distribution of multigenic family members responding to VHSV across L1, L2 and L3 lists.

Additional file 11. Figure S3—Single cell heatmaps of L2 and L3 gene lists. Heatmaps of L2 (B57) and L3 (AP2) genes from Table 2 identified from RNA-seq analysis represented on the scRNA-seq dataset. Genes were filtered for counts above 10 and then the average expression of each cell type was plotted. Gene numbers in brackets below the heatmap headers correspond to the numbers of genes found in the scRNA-seq/RNA-seq datasets.

Additional file 12. Table S8—Single cell transcriptomic analyses following VHSV infection.

## Data Availability

The RNA-Seq and scRNA-Seq data generated in this study have been submitted to the NCBI BioProject databases (https://www.ncbi.nlm.nih.gov/bioproject/). The RNA-seq data can be accessed under Bioproject accession number PRJEB57948 ([https://www.ncbi.nlm.nih.gov/bioproject/?term=PRJEB57948/](https:/www.ncbi.nlm.nih.gov/bioproject/?term=PRJEB57948), [82]) and the scRNA-seq data can be accessed under Bioproject accession number PRJNA1261575 ([https://www.ncbi.nlm.nih.gov/bioproject/PRJNA1261575/](https:/www.ncbi.nlm.nih.gov/bioproject/PRJNA1261575), [83]).

## Abbreviations

Adjp: Adjusted *P*-value
Ag: Antigen
cDC: Conventional dendritic cells
CyHV3: Cyprinus herpesvirus type 3
FC: Fold change
GO: Gene Ontology
IFN: Interferon
Ig: Immunoglobulin
iNKT: Invariant natural killer T cells
IPNV: Infectious pancreatic necrosis virus
ISG: Interferon-stimulated gene
MHC: Major histocompatibility complex
MNP: Mononuclear phagocytes
MoC: Monocytic cells
PBL: Peripheral blood leucocyte
PCA: Principal component analysis
QTL: Quantitative trait loci
scRNA-seq: Single-cell RNA sequencing
TCR: T-cell receptor
TRAV: T-cell receptor alpha variable genes
WOAH: World Organization for Animal Health
VHSV: Viral hemorrhagic septicemia virus
