# Supplementary material for: Immune cell-resolved transcriptomics provides insights into the basis for variations of fish genetic resistance to viral disease
Source: BMC Biol. 2025 Nov 25;23:348. doi: 10.1186/s12915-025-02452-z (PMC12648952; doi:10.1186/s12915-025-02452-z)
Supplement: Supplementary file 5 — Additional file 5. Further analysis of single cell data of T cells, B cells, Monocytes, Erythroid cells, HSC-Like, Thrombocytes and NK-Like, and code used for single cell analysis. [file 12915_2025_2452_MOESM5_ESM.docx]

**Additional File 5:**

**Immune cell-resolved transcriptomics provides insights into the basis for variations of genetic resistance to viral disease**

Thomas Clark *et al*.

Corresponding authors: Pierre Boudinot, pierre.boudinot@inrae.fr; Sam Martin, sam.martin@abdn.ac.uk

**The PDF file includes:**

Supplementary Single Cell Analysis

Supplementary Code

**Supplementary Single Cell Analysis**

***T-Cells***

Head kidney CD4+ cells represented a majority of our T-Cell populations (Figure 1), which could be further divided into four subclusters: “CD4+” and “CD4+ proliferating” identified by high expression of proliferating markers such as pcna, mcm6 and top2a; “T-Reg” defined by the expression of homologs of the classical specific markers foxp3, il21r and ikzf4; and a small subcluster we named “Invariant” due to the quasi-exclusive expression of 2 closely related T Cell Receptor Alpha Variable genes (TRAV2-2 and TRAV2-3, [24], suggesting the expression of an invariant TCR. Head kidney “CD8+” cells were defined by a strong expression of both cd8a and cd8b, and by an enrichment in cells expressing typical genes associated with cytotoxicity such as perforins (prf1 and prf1.1) and crtam, reinforcing their cytotoxic function. A further cluster expressed even higher levels of crtam, perforins and the autophagy regulator mccc1 but without co-expression of CD4 or CD8 was identified as “CD4-CD8- cytotoxic”. This was consistent with previously reported observations in Atlantic salmon head kidney [25]. “CD4-CD8- cytotoxic” was also the only subcluster in which an expression of *tcr d* (delta) was detected, although by a low percentage of cells. However, no significant expression of *sox13* or *tcr* g (gamma) transcript was found, calling into question the idea that this cluster could contain a fraction of gd T cells. A few CD4-CD8- double negative cells formed a last tiny cluster expressing gata2, suggesting they might be iNKT cells (See Additional File 4-e for full list of subcluster markers).

We then performed a pseudo-bulk differential analysis between all AP2 and B57 T cells (Figure 1). This global approach pointed to several genes identified previously by the differential analysis of all head kidney cells: rpl37, rap1b, tspan7b, rac2 being enriched in AP2 cells while rps2.1, mhc1uka, B2m.1 and eef1da were more expressed in B57 cells, indicating these genes are consistently differential between the two fish lines rather than cell specific. Additional genes identified by the pseudo-bulk comparison comprised a homolog of samd9l (ENSOMYG00000067209), which is a typical ISG with antiviral activity. Human SAMD9 and SAMD9L are ACNases that specifically deplete phenylalanine tRNA (tRNAPhe), leading to codon-specific ribosomal pausing and antiviral activity, while being latent in non-infected cells [26]. Importantly, key residues binding tRNAPhe (K198, K214, and R221) are conserved between ENSOMYG00000067209 and the human SamD9/L; a tetraspanin-like gene; two heat shock protein genes (hsp70a, and hsp90aa1.2.1); two additional mhc1 genes, uka and OnmyUAA-OSU; and ICN (ictacalcin) made up the top 10 most differential genes of B57 T-Cells. Other interesting genes significantly different included: TAGAP (T-cell activation RhoGTPase-activating protein), the fish specific interferon regulatory factor (IRF10) and an enhanced signature of activation induced cell death, the down phase of an immune response necessary to maintain immune homeostasis, with significantly higher expression of FOSB and the transcription factor JUN [27]. In contrast, AP2 T-Cells displayed higher expression of the atypical nuclease endod1 (ENSOMYG00000010767) that functions with cGAS-STING in innate immunity; TCR-beta (constant); FOSAB a homolog of FOSB; the sodium voltage-gated channel beta subunit 2 (scn2b); dnase1l4.1.1 and cysteine rich protein 2 (CRIP2) (See Additional File 4-c for full list of subcluster pseudobulk analysis).

Pairwise differential analyses across subclusters identified a possible enhanced immune phenotype within B57 T-Cells (Figure 1). CD2, a cell surface glycoprotein and IRF4 were upregulated across several B57 subsets with highest expression in CD8+ cells. CD2 has known roles in intracellular signalling with other antigen presenting cells, while IRF4, part of the interferon regulatory factor family, is induced following TCR stimulation and plays a role in the activation of CD8+ T-Cells [28, 29]. CRTAM and two paralogs of the TLR-inducible gene ZC3H12A an RNase essential for controlling immune responses by regulating mRNA decay [30], were upregulated in both CD8+ and CD8- cytotoxic subsets, with one paralog displaying differential expression and limited to only CD8+. Tumor necrosis factor receptor superfamily member, TNFRSF9a was exclusively expressed by CD8- cytotoxic cells and has roles in mammalian T-Cells in T-Cell development and proliferation. Another member of the IRF family, this time a fish specific member IRF10 [31] and TCRalpha constant were highest expressed in both CD8- cytotoxic and T-Regs. Vamp8, the human ortholog contributes to the TRIM6-Mediated type I interferon antiviral response [32] and IL10RA encoding the receptor for anti-inflammatory cytokine IL10 showed the highest expression in our invariant B57 population. While in AP2 cells, many of the most significant and specific genes were detected in CD4+ proliferating cells and included LSM8, a protein that plays a role in pre-mRNA splicing; molecular chaperone and mediator of glycoprotein folding PDIA3; and ribonucleoprotein NOP58. Interestingly, PSMB8 was also highly and specifically expressed in this proliferating population. Other genes included interleukin-2-inducible T-cell kinase gene (ITK) and IL20RA with roles in both pro and anti-inflammatory responses were mist expressed in AP2 CD8+ T-Cells. Finally, the highly specific expression of the CC chemokine in a significant fraction of CD4-CD8-cytotoxic cells could suggest a possible difference of chemotaxis (See Additional File 4-f for full list of subcluster pairwise analysis).


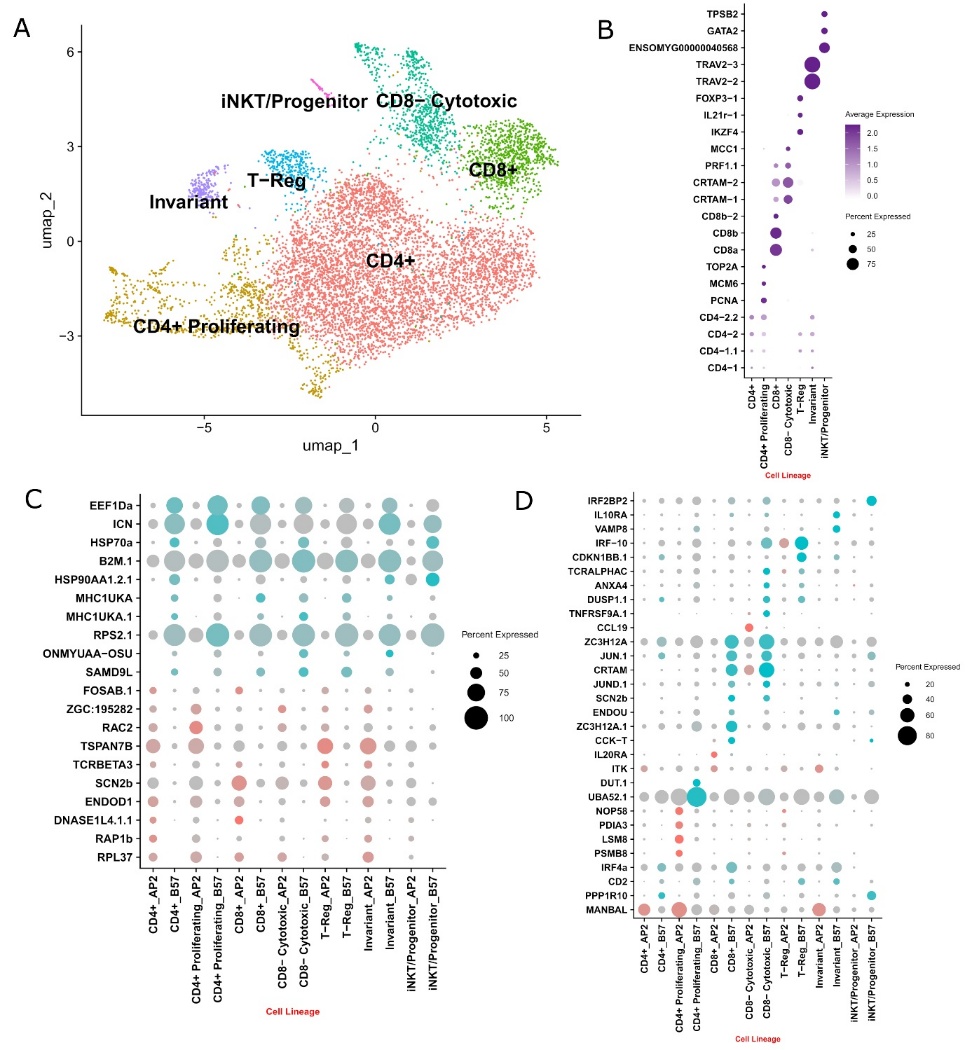


Figure 1. T-Cells

A. UMAP of T-Cell populations represented by different colours. B. Dot plot of T-Cell marker genes for each identified subtype. The size of the dots corresponds to the percentage of cells in a cluster expressing each marker indicated by the legend on the right of the plot, while colour intensity represents level of expression. C. Dot plot of top differential genes identified from pseudobulk analysis of all T-Cell populations (AP2 vs B57). D. Dot plot of top differential genes for each T-Cell subtype identified from pairwise comparisons AP2 vs B57. Colour intensity represents level of expression in AP2 (red) and B57 (blue) scaled by gene, grey colour represents lower expression

***B-Cells***

The largest cluster “B Cells” displayed no highly specific markers; genes add3, cd83 and blnk being the best identifiers of this cluster (Figure 2). A precursor subcluster “Pre-B Cells” was identifiable by the highly specific expression of the two terminal deoxy transferases likely involved in the VDJ rearrangement process, and by a key transcriptional regulator for pre B-Cell development, GFI1ab [33]. Another subcluster termed “Transitional” was characterized by the specific expression of S100A10a, CKBA and PLEK.1. In human plek-1 is exclusively expressed in several types of leukocytes and plays a role in cell spreading, stimulation and reorganization of the cytoskeleton [34]. This was consistent with the specific expression of S100A10a, another gene involved in leukocyte activation. Two additional clusters of cycling cells: “In-Cycle (G1/S)” and “In-Cycle (G2/M)” were identified by their high levels of cycling and proliferation genes, but with very clear differences in cell cycle stage respectively indicated by the associated markers [35]. Lastly, a subset of “Plasma B Cells” identified by very high levels of both heavy (m) and light chain Ig transcripts. Cells in this subcluster also show a highly specific expression of Thioredoxin Domain Containing (TXNDC)-5 and Peroxiredoxin (PRDX)-4, for which there was no obvious link with plasma cell biology (Additional File 4-e).

A pseudobulk analysis of AP2 and B57 B cells pointed to several genes already captured from the differential analysis of all head kidney cells: rpl37, rpl30.1, hmgb2a.1, rps2.1, eef1da, b2m.1 and mhc1uka (Figure 2). Many of the differences between AP2 and B57 B cells involved genes related to antigen presentation such as the two mhc1uka paralogs, or b2m.1 with higher expression across all B57 subsets. In contrast, other transmembrane related genes showed higher expression in most AP2 B-cell subclusters, included three paralogs of the glycoprotein CD9 (a1 and a2), and BCR components CD79a and CD79a.1. Further differences could also be observed between many genes encoding both variable and constant domains of lambda light chains with gene expressing the largest differences: igic1s1.1, igic1s1.2, igic1s1.3, igic1s3 and igl3v5 highlighted in the dotplot (Additional File 4-c).

We then performed pairwise differential analyses across subclusters (Figure 2; Additional File 4-f). Two genes were differential between AP2 and B57 “B-Cells” subclusters: A RAP1B paralog higher in B57 and IFI30 (or GILT), which is typically omnipresent in antigen presenting cells and plays an important role in MHC class II antigen processing [36] was more expressed in AP2 particularly in transitional/Activated, and In -Cycle subclusters; In “Pre B-Cells”, the transcriptional repressor bcl6aa that has important roles in innate and adaptive immunity was highly expressed in B57 fish. Two paralogs of GRAP, an important adaptor protein in signal transduction in leukocytes and positively correlated with infiltration of many immune cells, was also found to be expressed at higher levels in a larger percentage of B57 Pre B-Cells, although is typically associated with T-lymphocytes in mammals [37]. Other genes without an obvious involvement in the immune response: capn1a, cck-t, flot2a and dkc1 were all expressed at a higher level in B57, while apoeb, ddit4.1 and krt18a.1 were more expressed in AP2. Many genes were found to be differential in both “In-Cycle” clusters, although unsurprisingly, these tended to be primarily involved in various process related to cellular proliferation, actin organisation and cellular metabolism. However, cd200, a gene involved in immunomodulation and antiviral responses in humans [38] had two differentially and specifically expressed paralogs in AP2 versus B57 In-Cycle G2/M cells. Finally, “Plasma B Cells” showed specific differences in expression between the two lines for the lectin lgals9l3 and another MHC gene (MHC2b) this time specific to AP2; while higher and very specific expression of two Phospholipase A And Acyltransferase 4 (plaat) genes with involvement in innate immunity and inflammation were observed in B57.


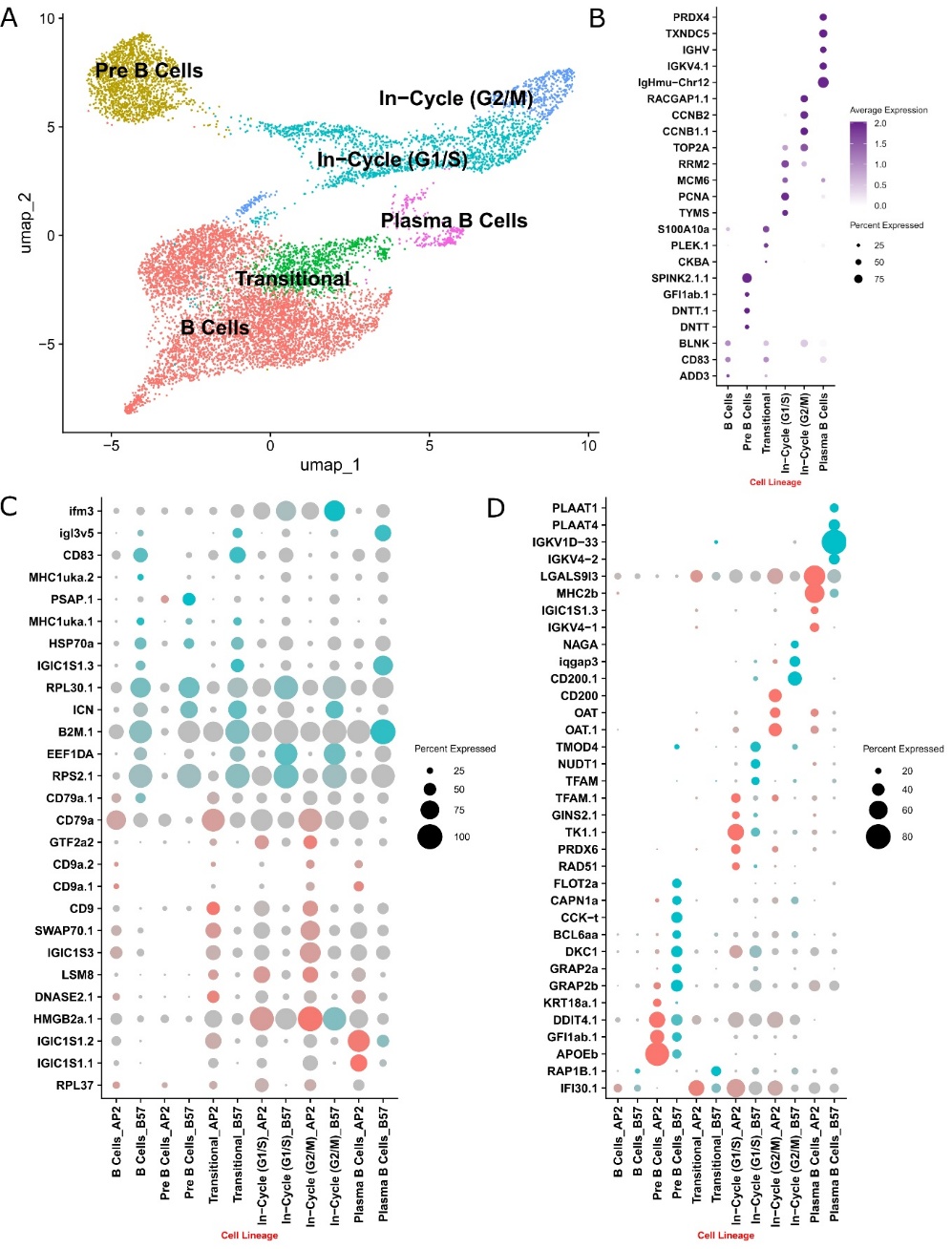


Figure 2. B-Cells

A. UMAP of B-Cell populations represented by different colours. B. Dot plot of B-Cell marker genes for each identified subtype. The size of the dots corresponds to the percentage of cells in a cluster expressing each marker indicated by the legend on the right of the plot, while colour intensity represents level of expression. C. Dot plot of top differential genes identified from pseudobulk analysis of all B-Cell populations (AP2 vs B57). D. Dot plot of top differential genes for each B-Cell subtype identified from pairwise comparisons AP2 vs B57. Colour intensity represents level of expression in AP2 (red) and B57 (blue) scaled by gene, grey colour represents lower expression

***Monocytes***

Reclustering of the myeloid cells excluding the polymorphonuclear leukocytes identified 9 clusters corresponding to mononuclear phagocytes (MNPs), that encompass monocytes, macrophages and dendritic cells in vertebrates [39] (Figure 3; Additional File 4-e). Based on top-expressed genes corresponding to canonical markers of MNP subsets in mammals, a first cluster can be assigned to conventional dendritic cells “cDC” with an enriched signature comprising IRF4, ID2, UNC, and importantly FLT3 (LOG2FC = 8.22; and 67% + cells versus 0,4% in other MNP) [39]. Interestingly, these cells also expressed high levels of IL12B, in a specific manner (LOG2FC= 8.26; and 45% positive cells versus 0,5% in other monocytes/macrophage subsets), as described in mice with a role in naive CD8+ T cell activation [40]. A second subset, “Alveolar Macrophage-like”, appears to align with macrophage types derived from embryonic progenitors such as the alveolar macrophages in mammals, with the dominant expression of MARCO and FABP4 gene(s), associated to high expression of C1Q, ID2, cathepsin and mpeg1. The remaining clusters correspond to monocyte-related cells (MoCs), as inferred from the enriched expression of typical monocytes markers such as CSF1R [41], NR4A1 [42], and C5AR1 [43]; however, based on this gene expression analysis, it was not possible to reliably classify these clusters as monocytes or macrophages, therefore we designate them as subsets of monocytic cells (MoCs). The “DC-SIGN+ MoCs” subset expresses high relative levels of CD209 (aka DC-SIGN) and CD83 as well as of genes involved in inflammatory responses, such as HMOX1 and TNFAIP3/A20(anti-inflammatory), IL8, IL1B and CCL4 (inflammatory), GPX1(redox control), suggesting their role in inflammation. DC-SIGN+ MoCs expressed several other markers suggesting a differentiation towards macrophages, including C1Q paralogs, 2 paralogs of lysozyme, MHC class 2 and CD74. We named another subset “CD11c+ MoCs”, as these cells displayed high expression of CSF2R and C1Q that usually corresponds to a macrophage differentiation in mammals [43]; of note, these CD11c+ MoCs also show high levels of UNC93B and IFITM1/IFITM3 mRNA, involved in IFN signaling, together with elevated expression of NRP1 and SLAMF8, that are hallmarks of plasmacytoid dendritic cell in mammals. Additionally, CD11c+ MoCs show high expression of genes involved in trafficking and phagocytosis [FCGR1A, a CD300-related regulatory factor]. Two clusters of “Proliferating MoCs” were also identifiable by their high levels of cell cycle genes and PCNA. High and specific expression of GATA2, a key transcription factor in the differentiation of hematopoietic lineages distinguished a subset of proliferating monocytes [44]. The other clusters stand as undefined MoCs.

Pseudobulk analysis of monocytes saw similar expression differences as previously seen between AP2 and B57 cells for RPL37, AEP1, CAPGB.1, RPS2.1, EEF1DA and RPL30.1 (Figure 3; Additional File 4-c). While other differential genes expressed across multiple monocyte sub lineages displayed a range of diverse function. Notably, expression of 2 genes from the 20S proteasome subunit beta family (PSMB8a and PSMB10) were highly expressed in several clusters of AP2 in contrast to B57 cells. Glioma pathogenesis-related 1 (GLIPR1), part of the CAP super family, whose members are mostly secreted with endocrine or paracrine functions in multiple physiological and pathological processes [27]. CD209-d, ICAM3 also with roles in immune responses were higher expressed across several AP2 monocyte subsets. In B57, Fc fragment of IgG receptor IIb shortened to FCGR2B, is typically highest expressed in B-Cells and certain populations of granulocytes although is still expressed at a lower level in monocytes. Co-engagement of FCGR2B with an activating type receptor such as FCGR2BA or the B cell antigen receptor modulates their ITAM-mediated activation signal pathway (Immunoreceptor Tyrosine-based Activation Motif), and expression on innate effector cells can modulates cell activation and antigen presentation [45]. Other immunity related genes included the chemokine CCL35.1 and MHC class I OnmyUAA-OSU.

Our pairwise comparisons of the monocytic cells identified a number of differences between our two isogenic lines, particularly in AP2 (Figure 3; Additional File 4-f). In GATA2+ proliferating MoCs (AP2), 3 paralogs and 1 further member of dual specificity protein phosphatase genes (DUSP5 and DUSP2) were all specifically expressed in a majority of these cells. Phosphorylation of serine, threonine, or tyrosine residues is a typical post translation modification that has critical function in signalling transduction pathways for many cellular processes including immunological signalling. DUSP proteins are able to dephosphorylate these residues acting as regulators; in humans, DUSP5 was induced following LPS stimulation in macrophage cells and was shown to act in an anti-inflammatory manner by inhibiting ERK and NF-κB signalling pathways [46]. In both B57 proliferating MoC clusters, specific expression of genes associated with DNA synthesis and repair: SEM1, TK1, TFAM and PCBD2 (GATA2+ only) was observed. Contrasted expression of two paralogs of DUT (Deoxyuridine Triphosphatase), an enzyme involved in nucleotide metabolism, was also seen in both proliferating cell clusters between AP2 and B57. The first signs of an enhanced immune signature within our subsets was seen in AP2 cells. Expression of two MHC class II genes (MHC2b and HLA-DQA1), CD99, galectin-3 binding protein LGALS3BPa, CD209d like (a different DC-SIGN paralog), the GTPase GVINP1 (very large interferon inducible pseudogene 1), CSF1ra and two paralogs of SOX9a (although increased expression was observed in B57 cDC cells) were all enhanced in various combinations of DC-SIGN, CD11c+ and Alveolar Macrophage like clusters. In B57, TRIM109 and FCRL5.2 (member of the immunoglobulin receptor superfamily) were more expressed in CD11c+ MoCs, while the proinflammatory interleukin (IL-8) was expressed in DC-SIGN MoCs.


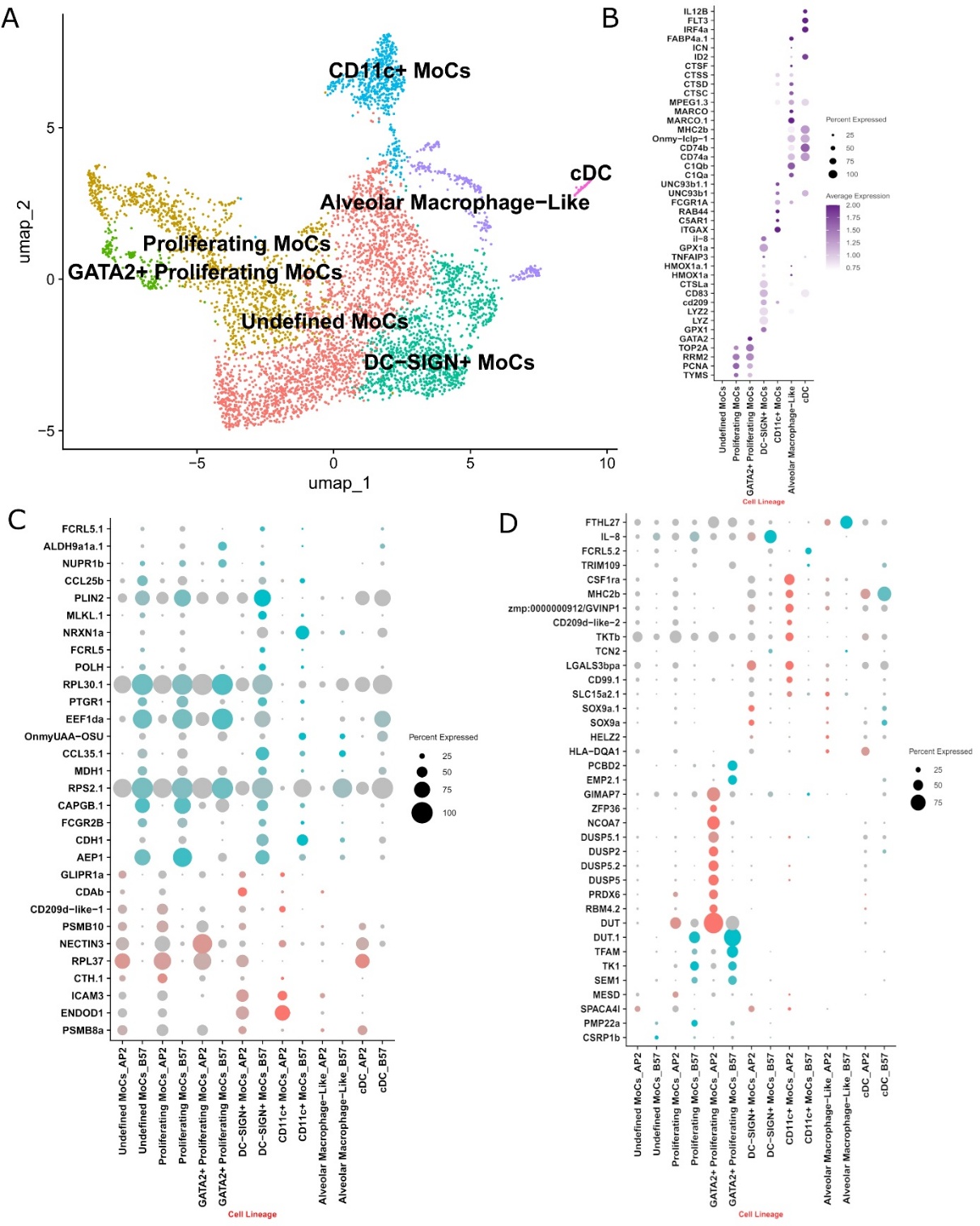


Figure 3. Monocytes

A. UMAP of Monocyte populations represented by different colours. B. Dot plot of Monocyte marker genes for each identified subtype. The size of the dots corresponds to the percentage of cells in a cluster expressing each marker indicated by the legend on the right of the plot, while colour intensity represents level of expression. C. Dot plot of top differential genes identified from pseudobulk analysis of all Monocyte populations (AP2 vs B57). D. Dot plot of top differential genes for each B-Cell subtype identified from pairwise comparisons AP2 vs B57. Colour intensity represents level of expression in AP2 (red) and B57 (blue) scaled by gene, grey colour represents lower expression

***Erythroid-Cells***

Despite processing head kidney tissue using a percoll gradient to extract leucocytes, we still recovered a significant population of red blood cells characterised by high levels of haemoglobin genes (Figure 4; Additional File 4-e). We chose to analyse these in the same way as our other leukocyte cell types by re-subsetting and clustering. Within our erythroid population, we were able to identify 6 distinct subpopulations: Erythroid-Cells formed the largest cluster and were named due to their lack of specific expression and presence of the largest proportion of haemoglobin genes. Three populations could be described as cell-cycling clusters: G1/S, G2/M and Intermediate by co-expression of both stages’ markers. Interestingly, two clusters of immune gene expressing Erythroid-Cells were also captured. These clusters were characterised by their expression of ISG15, PRSS16 and expression or lack of expression of VIG1. This is not the first-time red blood cells in fish have been described to have an immune phenotype. ISAV is one virus that is able to infect red and white blood cells in fish; following an infection in Atlantic salmon, type I and II interferon inducible genes, Mx and γIP were found to be upregulated in red blood cells [47].

Pseudobulk analysis in AP2 Erythroid-Cells did not capture any of the same prevalent genes we previously observed in our global comparison, while in B57: RPS2.1, B2m.1 and EEF1Da were found again in their Erythroid population, perhaps unsurprisingly, B2M.1 expression was highest in our B57 immune related clusters. Interestingly, several immune genes with highly differential expression were detected in both AP2 and B57 immune related Erythroid populations. In AP2 two paralogs of LYG, a lysozyme involved in innate immunity against bacteria that kills bacteria through hydrolysis of cell wall peptidoglycan [48]; ICAM3; A paralog of SAMD9L, whose other paralog was previously observed with higher expression in B57 cells; and PSMB10 implicated in antigen presentation and antiviral immunity. In contrast, the only directly immunity related gene detected in B57 bar B2M was CD99 (Additional File 4-c).

Pairwise comparisons of our Erythroid subsets revealed two contrasted groups from the subsets: cycling Erythroid-Cells (Intermediate Cell Cycle and the two In-Cycle clusters G1/S,G2M) and the immune gene expressing Erythroid-Cells, mainly the Immune I specific cluster. For the cycling Erythroid-Cells, AP2 cells predominantly showed higher expression of COX8A, PSMB8, RPL37, PONZR1, CHST1 at the G1/S state, although much lower expression could still be seen in the other cycling clusters. While across all AP2 cycling clusters, the cellular adhesion molecule NECTIN3 and carbohydrate sulfotransferase enzyme (CHST12a) were much higher expressed. B57 cycling Erythroid showed fairly similar expression of their differential genes across the three stages. These included numerous enzymes (GPT.1, DUT.1, TALDO, HPD and ALDH9a1a.1) involved in nucleotide or amino acid metabolism, a fatty acid binding protein (FABP3) and a small heat shock protein (CRYAB) part of the HSP20 family. CD200 was once again seen in an AP2 G2M cycling cluster specifically expressed (see B-Cell analysis for other expression). In “Immune VIG1” expressing cells, AP2 clusters showed higher expression of another haemoglobin gene (HBE1) an aquaporin (AQP1a.1) and the ATP-dependent RNA helicase DHX37. B57 immune cells however showed increased expression of two RPL genes, a ubiquitin like protein (UBLP) as well as the ISG PARP12 and VIG1 (Additional File 4-f).

***
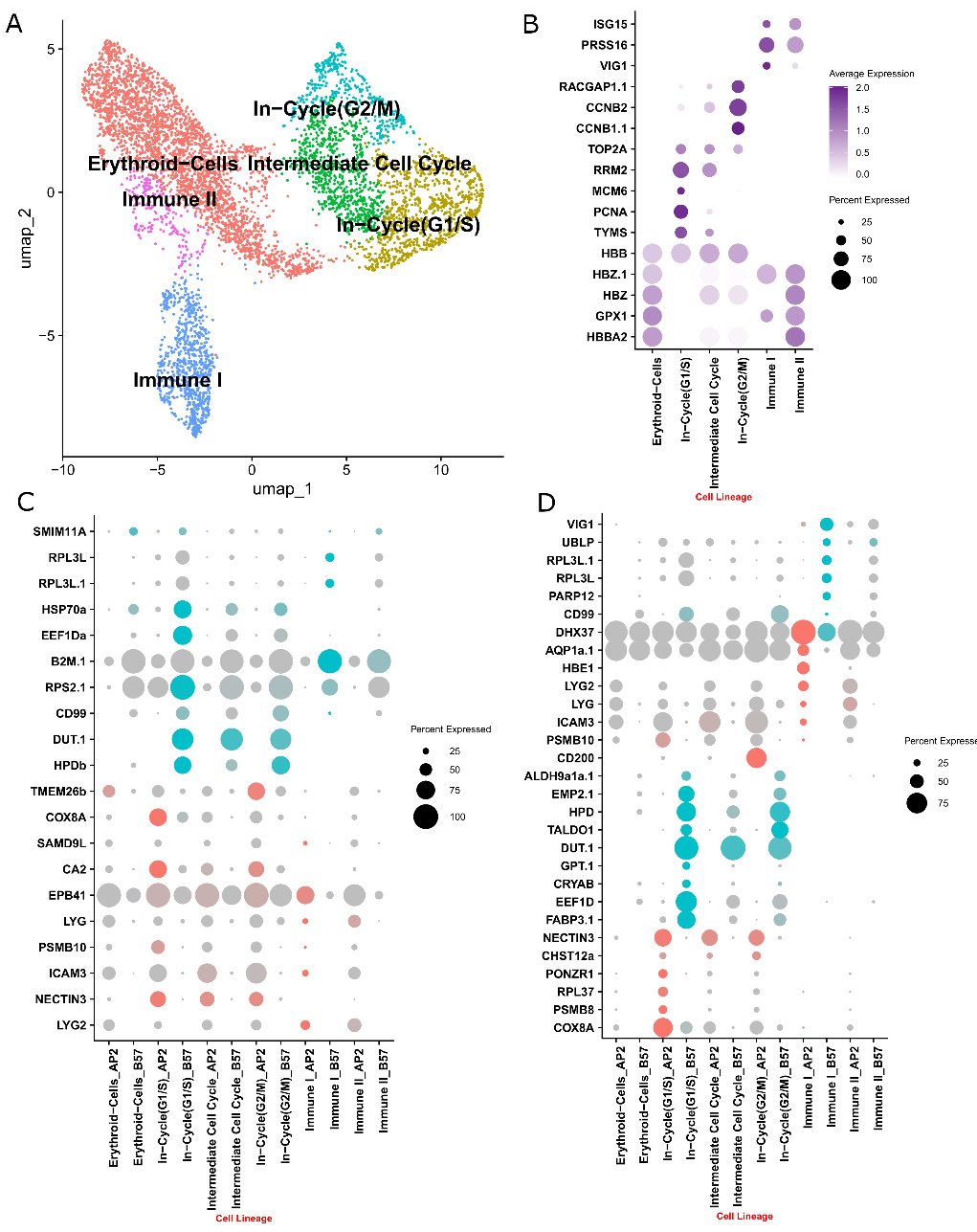
***

Figure 4. Erythroid-Cells

A. UMAP of Erythroid populations represented by different colours. B. Dot plot of Erythroid marker genes for each identified subtype. The size of the dots corresponds to the percentage of cells in a cluster expressing each marker indicated by the legend on the right of the plot, while colour intensity represents level of expression. C. Dot plot of top differential genes identified from pseudobulk analysis of all Erythroid populations (AP2 vs B57). D. Dot plot of top differential genes for each Erythroid subtype identified from pairwise comparisons AP2 vs B57. Colour intensity represents level of expression in AP2 (red) and B57 (blue) scaled by gene, grey colour represents lower expression

***3.2.7 HSC-Like, Thrombocytes and NK-Like***

The remaining clusters only comprised 4.3% of the total cells in our experiment and were deemed too small for further subsetting and re-clustering, instead were only analysed by pseudobulk analysis, AP2vsB57 (Figure 5; Additional File 4-c). While there were no major differences in the proportion of cells between these lineages, differences in gene expression identified several genes already captured throughout the analysis. In AP2, clusters displayed similar expression in RPL37 but only in HSC-Like, RAP1B and TSPAN7b were most expressed in thrombocytes while PRDX5.1. was less expressed in thrombocytes but highest in NK-Like cells. In B57 lineages, RPS2.1 and EEF1Da was most expressed in HSC-Like; CAPGB.1 and B2M.1 in thrombocytes, but some expression in HSC-like; two paralogs of MHC1UKA were highest expressed in B57, but with differential cell type expression with one paralog highest in NK-Like and the other in thrombocyte cells. Our differential pairwise analysis also identified numerous other genes, with various functions in physiological process and several genes linked with immune function. Surprisingly, we found two antiviral genes PSMB8a and transmembrane protein IFITM1 highest expressed in AP2 HSC-Like cells. While HSC cells are not thought as directly linked with immune responses, they have been viewed as having a reactive homeostatic response to replenish immune cells during an infection by direct cytokine signalling, in lieu of sensing a depletion of downstream cells [49]. B57 HSC-Like cells displayed no similar specific phenotype. The inflammatory interleukin IL8b was specifically expressed in AP2 thrombocytes, but is typically expressed by many immune cell types; platelets have IL8 receptors and react with potent procoagulant activity upon activation [50]. Another potentially pro-coagulation linked gene AQP1A.2 was also higher expressed in AP2, aquaporins typically mediate the conduction of water across cell membranes and water entry promotes procoagulant activity in platelets [51], indicating a potentially improved clotting function in AP2 fish. While in B57 thrombocytes, IL17Rb another proinflammatory cytokine receptor and NDRG2 were specifically and highest expressed. Adenovirus-mediated N-Myc downstream-regulated gene 2 (NDRG2) has been linked with inhibitory effects on proliferation of cancerous cells, tumor growth and metastasis [52]. In our NK-like lineage, PRDX5 which functions as an antioxidant to prevent cellular damage from peroxides, PRF1.3 and two paralogs of ART were more expressed in AP2. Interestingly, one of our marker genes for NK-like cells MCCC1, also observed in our CD8- cytotoxic T-Cell population, was higher expressed in AP2 compared to B57, although still detected. In contrast, paralogs of perforin (PRF1) and MHC class I (ONMYUAA-OSU), SAMD9L and proinflammatory cytokine IL12B were all higher and specifically expressed potentially pointing to an enhanced cytotoxic profile in B57 NK-Like cells.

**Supplementary Code**

**#Code used for head kidney single cell analysis**

**#Install and load packages**

install.packages("dplyr")

install.packages("Seurat")

install.packages("patchwork")

install.packages ("multtest")

install.packages ("metap")

install.packages ("ggplot2")

install.packages("scater")

install.packages("loomR")

install.packages("Seurat")

install.packages("scDblFinder")

install.packages("monocle")

install.packages("SingleCellExperiment")

install.packages("ggpubr")

install.packages("harmony")

install.packages("sctransform")

install.packages("scCustomize")

install.packages("dittoSeq")

install.packages("viridis")

library(dplyr)

library(Seurat)

library(patchwork)

library (multtest)

library (metap)

library (ggplot2)

library(scater)

library(loomR)

library(Seurat)

library(scDblFinder)

library(monocle)

library(SingleCellExperiment)

library(ggpubr)

library(harmony)

library(sctransform)

library(scCustomize)

library(dittoSeq)

library(viridis)

**#Reading count matrix into seurat and removing doublet cells computationally**

**#HKA1**

hka1.data <- Read10X(data.dir ="PATH")

hka1 <- CreateSeuratObject(counts = hka1.data, project = "hka1", min.cells = 3, min.features = 100)

hka1.sce <- as.SingleCellExperiment(hka1)

hka1.sce.dbl.out <- scDblFinder(hka1.sce, clusters=FALSE)

hka1 <- as.Seurat(hka1.sce.dbl.out, counts = "counts", data = "logcounts")

hka1 = hka1[, hka1$scDblFinder.class == "singlet"]

**#HKA2**

hka2.data <- Read10X(data.dir ="PATH")

hka2 <- CreateSeuratObject(counts = hka2.data, project = "hka2", min.cells = 3, min.features = 100)

hka2.sce <- as.SingleCellExperiment(hka2)

hka2.sce.dbl.out <- scDblFinder(hka2.sce, clusters=FALSE)

hka2 <- as.Seurat(hka2.sce.dbl.out, counts = "counts", data = "logcounts")

hka2 = hka2[, hka2$scDblFinder.class == "singlet"]

**#HKB1**

hkb1.data <- Read10X(data.dir ="PATH")

hkb1 <- CreateSeuratObject(counts = hkb1.data, project = "hkb1", min.cells = 3, min.features = 100)

hkb1.sce <- as.SingleCellExperiment(hkb1)

hkb1.sce.dbl.out <- scDblFinder(hkb1.sce, clusters=FALSE)

hkb1 <- as.Seurat(hkb1.sce.dbl.out, counts = "counts", data = "logcounts")

hkb1 = hkb1[, hkb1$scDblFinder.class == "singlet"]

**#HKB2**

hkb2.data <- Read10X(data.dir ="C:/Users/thoma/Desktop/Single cell

analysis/results/hkb2/hkb2_filtered_rescue1/")

hkb2 <- CreateSeuratObject(counts = hkb2.data, project = "hkb2", min.cells = 3, min.features = 100)

hkb2.sce <- as.SingleCellExperiment(hkb2)

hkb2.sce.dbl.out <- scDblFinder(hkb2.sce, clusters=FALSE)

hkb2 <- as.Seurat(hkb2.sce.dbl.out, counts = "counts", data = "logcounts")

hkb2 = hkb2[, hkb2$scDblFinder.class == "singlet"]

**#Assinging meta data to the samples**

hka1@meta.data [["Line"]] <- (ident = "AP2")

hka2@meta.data [["Line"]] <- (ident = "AP2")

hkb1@meta.data [["Line"]] <- (ident = "B57")

hkb2@meta.data [["Line"]] <- (ident = "B57")

hka1@meta.data [["Tissue"]] <- (ident = "HK")

hka2@meta.data [["Tissue"]] <- (ident = "HK")

hkb1@meta.data [["Tissue"]] <- (ident = "HK")

hkb2@meta.data [["Tissue"]] <- (ident = "HK")

hka1@meta.data [["Condition"]] <- (ident = "Control")

hka2@meta.data [["Condition"]] <- (ident = "Control")

hkb1@meta.data [["Condition"]] <- (ident = "Control")

hkb2@meta.data [["Condition"]] <- (ident = "Control")

**#merge all seurat objects into one**

hk <- merge(hka1, y = c(hka2,hkb1,hkb2), add.cell.ids = c("hka1","hka2","hkb1","hkb2"), project = "HK")

saveRDS(hk, file = "PATH/HK_Control_Initial_Analysis.rds")

**#Subset the data based on number of genes detected per cell, I've lowered and increased the max min values compared to default seurat settings. Extremely low numbers of features indicate empty GEMs and excessive gene numbers could indicate doublets**

hk<- subset(hk, subset = nFeature_RNA > 200 & nFeature_RNA < 3000)

**#Standard seurat workflow aside from the inclusion of RunHarmony to deal with batch effects, number of dimensions to use in "RunUMAP" and "FindNeighbours" is assessed from elbow plot.**

hk <- NormalizeData(hk, normalization.method = "LogNormalize", scale.factor = 10000)

hk <- FindVariableFeatures(hk, selection.method = "vst", nfeatures = 2000)

all.genes <- rownames(hk)

hk <- ScaleData(hk, features = all.genes)

hk <- RunPCA(hk, features = VariableFeatures(object = hk))

ElbowPlot(hk, ndims=50)

hk <- RunHarmony(hk, group.by.vars = "orig.ident")

hk <- RunUMAP(hk,reduction = "harmony", dims = 1:21)

hk<- FindNeighbors(hk,reduction = "harmony", dims = 1:21)

hk<- FindClusters(hk, resolution = 0.8)

DimPlot(hk, reduction = "umap", label=T)

**#Find markers for all cell types, default seurat settings used then filtered manually in excel**

hk.markers <- FindAllMarkers(seurat.object, only.pos = TRUE, min.pct = 0.25, log2fc.threshold = 0.25)

**#Cannonical dotplot markers to aide assigning identies to clusters**

DotPlot(hk,cols = c("white","darkorchid4"), scale = T, col.min = -0.1, col.max = 3,dot.min = 0.05, dot.scale =

7, features = c("lect2","mpx.1", "mmp9",

"cd79a","ENSOMYG00000020682", "IgHmu-Chr12",

"ENSOMYG00000041305","ENSOMYG00000007053","ENSOMYG00000046200","cd8a",

"HBB","ENSOMYG00000035352",

"timp2b","cd209","lyz2", "mpeg1.3",

"myb","ENSOMYG00000023393", "ENSOMYG00000022982",

"ENSOMYG00000011383","TAGLN",

"prf1.3","aqp1a.2","ENSOMYG00000025718")) +

scale_x_discrete(labels = c("LECT2l","MPX","MMP9",

"CD79a","IGl4v8","IGM",

"IL7r","CCR7","CD3e","CD8a",

"HBBA1","HBBA2",

"TIMP2b","CD209","LYZ","MPEG",

"MYB","sb:cb81", "NPM",

"ITGB3b","TAGLN2",

"PRF1","AQP1A","MCCC1")) +

labs(x = "", y = "Cell Lineage") +

theme(axis.title.x = element_text(face="bold", colour="red", size="8")) +

theme(axis.text.x= element_text(face="bold", colour="black", size="10")) +

theme(axis.text.y= element_text(face="bold", colour="black", size="10"))+

theme(legend.text = element_text(size = 8), legend.title = element_text(size = 8)) + coord_flip() +

theme(axis.text.x = element_text(angle = 90, hjust=1))

**#Pseudobulk analysis, cell types are grouped into AP2 or B57 and then find markers ran to find differential genes**

seurat.object <- hk

seurat.object$sample.subtypes <- paste(Idents(seurat.object), seurat.object$Line, sep = "_")

seurat.object$celltype <- Idents(seurat.object)

Idents(seurat.object) <- "sample.subtypes"

DimPlot(seurat.object, reduction = "umap", label=T, split.by = "Line")

new.cluster.ids <- c("All_AP2", "All_AP2", "All_AP2", "All_AP2", "All_AP2", "All_AP2", "All_AP2", "All_AP2", "All_AP2", "All_B57", "All_B57", "All_B57", "All_B57", "All_B57", "All_B57", "All_B57", "All_B57", "All_B57")

names(new.cluster.ids) <- levels(seurat.object)

seurat.object <- RenameIdents(seurat.object, new.cluster.ids)

DimPlot(seurat.object, reduction = "umap", label=T)

**#AP2 vs B57 all cells, default seurat settings used then filtered manually in excel**

hk.markers <- FindAllMarkers(seurat.object, only.pos = TRUE, min.pct = 0.25, log2fc.threshold = 0.25)

**###########################################################################**

**#Example of Cell type analysis, cells are initially subset and then reanalysed using Seurats pipeline. Sub cell type identies are then assigned based on cannonical markers and those generated from "FindAllMarkers" function. Cell types are then compared at a whole lineage level AP2 vs B57, and then at the pairwise level between sub-cell types (AP2 vs B57)**

**#T-Cells**

tcell <- subset(x = hk, idents = "T-Cells")

DimPlot(tcell, reduction = "umap", label=T,label.size=10)

tcell <- NormalizeData(tcell , normalization.method = "LogNormalize", scale.factor = 10000)

tcell <- FindVariableFeatures(tcell , selection.method = "vst", nfeatures = 2000)

all.genes <- rownames(tcell)

tcell <- ScaleData(tcell , features = all.genes)

tcell <- RunPCA(tcell , features = VariableFeatures(object = tcell ))

ElbowPlot(tcell , ndims=50)

tcell <- RunHarmony(tcell, group.by.vars = "orig.ident")

tcell <- RunUMAP(tcell,reduction = "harmony", , dims = 1:21)

tcell<- FindNeighbors(tcell,reduction = "harmony", dims = 1:21)

tcell<- FindClusters(tcell, resolution = 0.4)

#finding markers for subsets

tcellmarkers<- FindAllMarkers(tcell, only.pos = TRUE, min.pct = 0.25, log2fc.threshold = 0.25)

**#assigning new cell IDs**

new.cluster.ids <- c("CD4+","CD4+","CD4+","CD8+","CD8- Cytotoxic","CD4+ Proliferating","CD4+ Proliferating", "T-Reg", "Invariant","CD4+ Proliferating_T-Cells","iNKT/Progenitor")

names(new.cluster.ids) <- levels(tcell)

tcell <- RenameIdents(tcell, new.cluster.ids)

DimPlot(tcell, reduction = "umap", label=T)

#Assigning Line data to each sub cell type

tcell$sample.subtypes <- paste(Idents(tcell), tcell$Line, sep = "_")

tcell$celltype <- Idents(tcell)

Idents(tcell) <- "sample.subtypes"

**#Pairwise comparisons default seurat settings used then filtered manually in excel**

**#CD4**

CD4.markers <- FindMarkers(object = seurat.object, ident.1 = "CD4+_AP2", ident.2="CD4+_B57")

**#CD4+ Proliferating_T-Cells**

CD4proliferating.markers <- FindMarkers(object = seurat.object, ident.1 = "CD4+ Proliferating_AP2", ident.2="CD4+ Proliferating_B57")

**#CD8**

CD8.markers <- FindMarkers(object = seurat.object, ident.1 = "CD8+_AP2", ident.2="CD8+_B57")

**#Invariant**

Invariant.markers <- FindMarkers(object = seurat.object, ident.1 = "Invariant_AP2", ident.2="Invariant_B57")

**#T-Reg**

Treg.markers <- FindMarkers(object = seurat.object, ident.1 = "T-Reg_AP2", ident.2="T-Reg_B57")

**#Cytotoxic**

cytotoxic.markers <- FindMarkers(object = seurat.object, ident.1 = "CD8- Cytotoxic_AP2", ident.2="CD8- Cytotoxic_B57")

**#iNKT**

iNKT.markers <- FindMarkers(object = seurat.object, ident.1 = "iNKT/Progenitor_AP2", ident.2="iNKT/Progenitor_B57")

**##Pseudobulk analysis T-cells**

seurat.object <-tcell

new.cluster.ids <-

c("All_AP2","All_AP2","All_AP2","All_AP2","All_AP2","All_AP2","All_AP2","All_B57","All_B57","All_B57", "All_B57", "All_B57", "All_B57", "All_B57")

names(new.cluster.ids) <- levels(seurat.object)

seurat.object <- RenameIdents(seurat.object, new.cluster.ids)

DimPlot(seurat.object, reduction = "umap", label=T,split.by="Line")

Tcellmarkers <- FindAllMarkers(seurat.object, only.pos = TRUE, min.pct = 0.25, log2fc.threshold = 0.25)
