# Supplementary material for: Immune cell-resolved transcriptomics provides insights into the basis for variations of fish genetic resistance to viral disease
Source: BMC Biol. 2025 Nov 25;23:348. doi: 10.1186/s12915-025-02452-z (PMC12648952; doi:10.1186/s12915-025-02452-z)

Figure S1 : Comparison of differentially expressed genes in AP2 and B57 head kidney transcriptomes after infection. DESEQ2 was used to compare B57 infected vs B57 control and AP2 infected vs AP2 control. Up-regulated genes were for FC >= 2 and padj <= 0.01, and down regulated genes for FC <= 0.5 and padj <= 0.01.

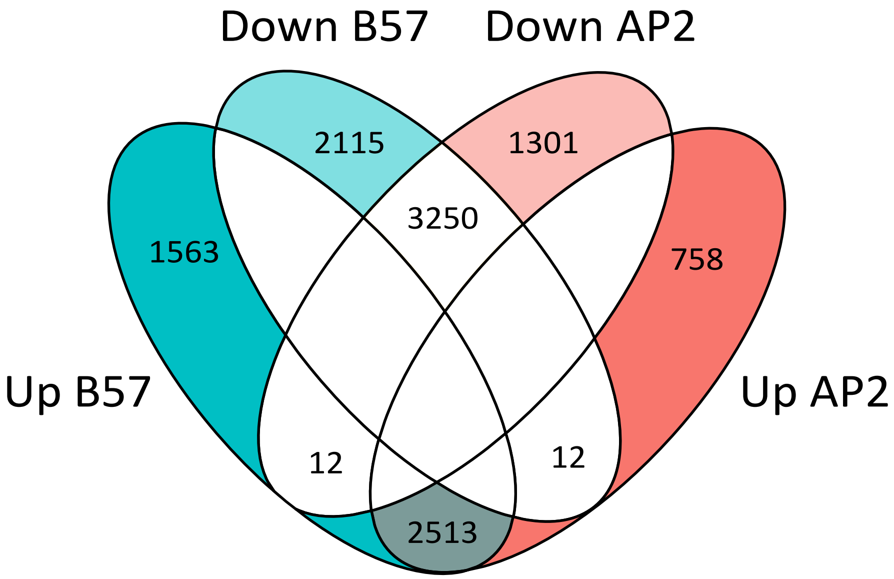

Supplement: Supplementary file 7 — Additional file 7. Figure S1- RNA-Seq Venn comparison of differentially expressed genes in AP2 and B57 head kidney transcriptomes after infection. [file 12915_2025_2452_MOESM7_ESM.pdf]
