# Supplementary material for: Immune cell-resolved transcriptomics provides insights into the basis for variations of fish genetic resistance to viral disease
Source: BMC Biol. 2025 Nov 25;23:348. doi: 10.1186/s12915-025-02452-z (PMC12648952; doi:10.1186/s12915-025-02452-z)

Figure S2. Quantification of VHSv G mRNA by RT-qPCR from spleen (S) and head-kidney (HK) of infected fish compared to controls.
Results are normalized to housekeeping genes beta-actin, rps29 and elf1alpha.-ΔCq values are shown. Given the high interindividual variation and the fact that the t-test value is close to the significance threshold ($p < 0.025$ after Bonferroni correction), the observed difference in G expression between AP2 and B57 spleen samples should be interpreted with caution.

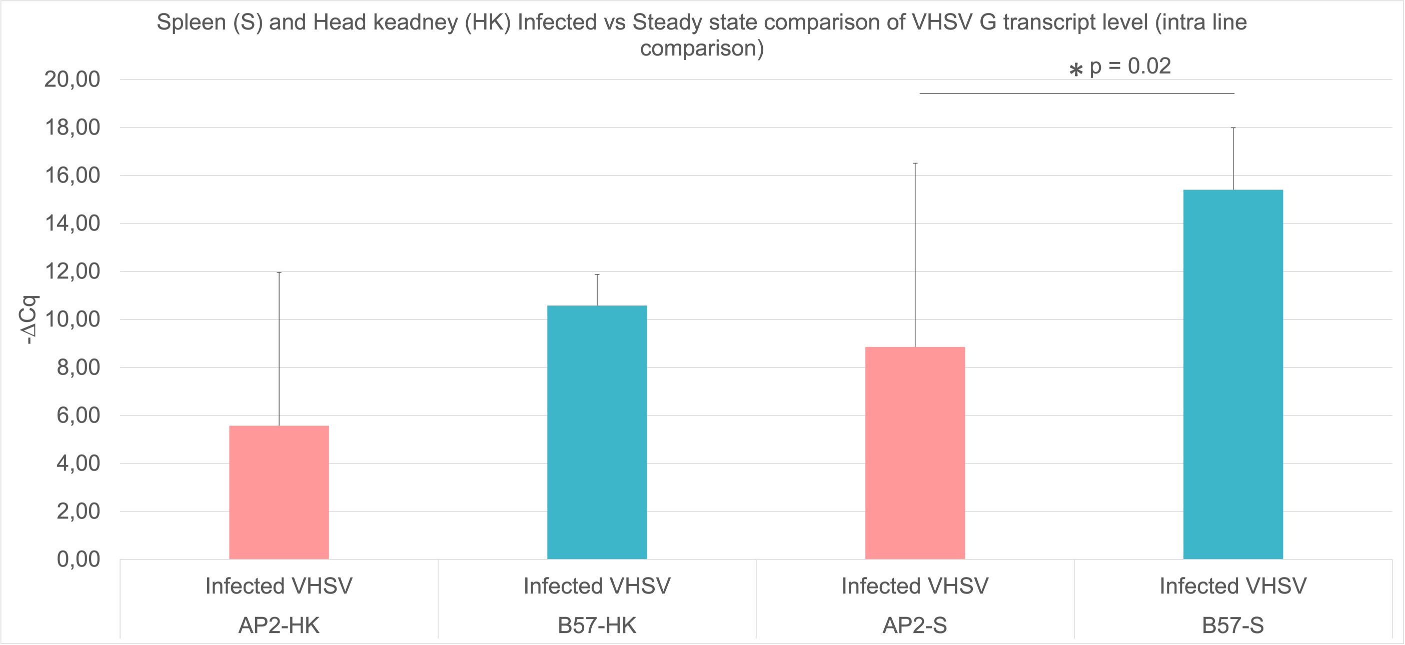

Supplement: Supplementary file 8 — Additional file 8. Figure S2—Quantification of VHSv G mRNA by RT-qPCR from spleen (S) and head-kidney (HK) of infected fish compared to controls. [file 12915_2025_2452_MOESM8_ESM.pdf]
