# Supplementary material for: Immune cell-resolved transcriptomics provides insights into the basis for variations of fish genetic resistance to viral disease
Source: BMC Biol. 2025 Nov 25;23:348. doi: 10.1186/s12915-025-02452-z (PMC12648952; doi:10.1186/s12915-025-02452-z)

Figure S3. Heatmaps of L2 (B57) and L3 (AP2) genes from Table 2 identifed from RNA-seq analysis represented on the scRNA-seq dataset. Genes were filtered for counts above 10 and then the average expression of each cell type was plotted. Gene numbers in brackets below the heatmap headers correspond to the numbers of genes found in the scRNA-seq/RNA-seq datasets. For gene lists see Table S8.

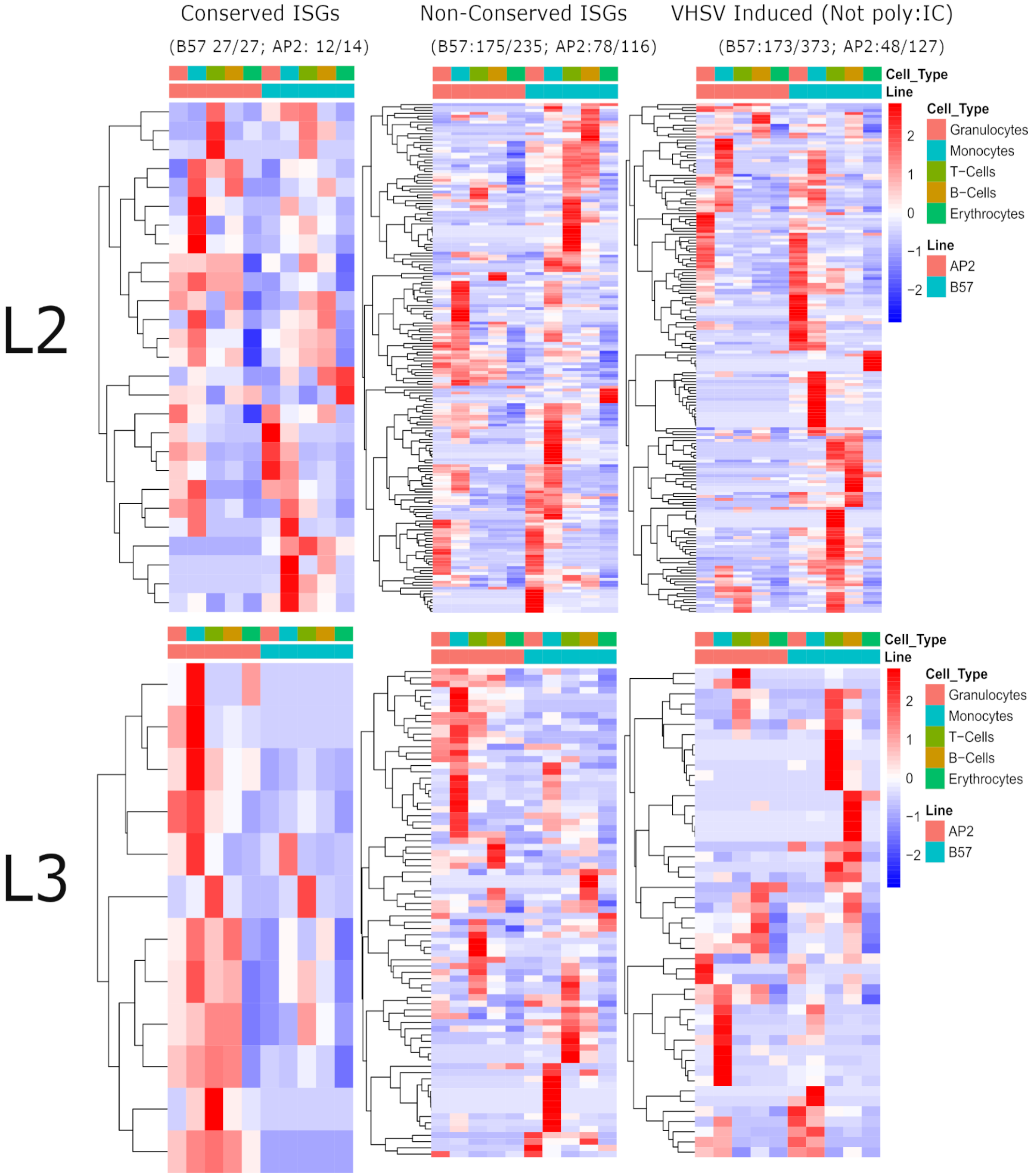

Supplement: Supplementary file 11 — Additional file 11. Figure S3—Single cell heatmaps of L2 and L3 gene lists. Heatmaps of L2 (B57) and L3 (AP2) genes from Table 2 identified from RNA-seq analysis represented on the scRNA-seq dataset. Genes were filtered for counts above 10 and then the average expression of each cell type was plotted. Gene numbers in brackets below the heatmap headers correspond to the numbers of genes found in the scRNA-seq/RNA-seq datasets. [file 12915_2025_2452_MOESM11_ESM.pdf]
